# The Phenolic compound Kaempferol overcomes 5-fluorouracil resistance in human resistant LS174 colon cancer cells

**DOI:** 10.1038/s41598-018-36808-z

**Published:** 2019-01-17

**Authors:** Ichrak Riahi-Chebbi, Soumaya Souid, Houcemeddine Othman, Meriam Haoues, Habib Karoui, Alain Morel, Najet Srairi-Abid, Makram Essafi, Khadija Essafi-Benkhadir

**Affiliations:** 1 Institut Pasteur de Tunis, LR11IPT04/LR16IPT04 Laboratoire d’Epidémiologie Moléculaire et de Pathologie Expérimentale appliquées aux Maladies infectieuses, 1002 Tunis, Tunisia; 2 Institut Pasteur de Tunis, LR11IPT08/LR16IPT08 Laboratoire des Venins et Biomolécules thérapeutiques, 1002 Tunis, Tunisia; 3 Institut Pasteur de Tunis, LR11IPT02/ LR16IPT02 Laboratoire de Recherche sur la Transmission, le Contrôle et l’Immunobiologie des Infections, 1002 Tunis, Tunisia; 4 CRCINA, INSERM U892, Université de Nantes, Université d’Angers, Paul Papin ICO cancer center, Angers, France; 5 Université de Tunis El Manar, 1068 Tunis, Tunisia

## Abstract

Resistance to 5-Fluorouracil chemotherapy is a major cause of therapeutic failure in colon cancer cure. Development of combined therapies constitutes an effective strategy to inhibit cancer cells and prevent the emergence of drug resistance. For this purpose, we investigated the anti-tumoral effect of thirteen phenolic compounds, from the Tunisian quince *Cydonia oblonga* Miller, alone or combined to 5-FU, on the human 5-FU-resistant LS174-R colon cancer cells in comparison to parental cells. Our results showed that only Kaempferol was able to chemo-sensitize 5-FU-resistant LS174-R cells. This phenolic compound combined with 5-FU exerted synergistic inhibitory effect on cell viability. This combination enhanced the apoptosis and induced cell cycle arrest of both chemo-resistant and sensitive cells through impacting the expression levels of different cellular effectors. Kaempferol also blocked the production of reactive oxygen species (ROS) and modulated the expression of JAK/STAT3, MAPK, PI3K/AKT and NF-κB. *In silico* docking analysis suggested that the potent anti-tumoral effect of Kaempferol, compared to its two analogs (Kaempferol 3-O-glucoside and Kampferol 3-O-rutinoside), can be explained by the absence of glucosyl groups. Overall, our data propose Kaempferol as a potential chemotherapeutic agent to be used alone or in combination with 5-FU to overcome colon cancer drug resistance.

## Introduction

Colorectal cancer (CRC) is one of the most frequently occurring malignancies worldwide^1^. According to GLOBOCAN data, there were over 1.8 million new colorectal cancer cases and 881,000 deaths in 2018, accounting for about 1 in 10 cancer cases and deaths^2^. Globally, colorectal cancer ranks third in terms of incidence but second in terms of mortality since 40–50% of patients develop metastatic disease (mCRC)^2,3^. Although several chemotherapeutic agents have been identified to improve survival and quality of life of CRC patients^4^, 5-Fluorouracil (5-FU) remains recommended as the drug of a first choice after more than 30 years of clinical research^5^. The antimetabolite drug elicits its cytotoxic effect mainly through inhibition of Thymidylate Synthase (TS), a key enzyme for catalyzing the novo synthesis of thymine^6^.

In CRC, 5-FU was used in monotherapy or in combination with oxaliplatin (Folfox), irinotecan (Folfiri), or irinotecan and bevacizumab (Folfiri-bevacizumab). Unfortunately, the adjuvant chemotherapeutic regimens rarely cure cancer and disease relapses from the drug-resistant cells^7^.

Thus, resistance, either intrinsic or acquired during the course of treatment, is a major challenge for cancer therapy^8^. The development of chemoresistance can be attributed to a wide variety of mechanisms including drug influx and efflux, enhancement of drug inactivation and mutation of the drug target^9^. Acquired 5-FU resistance is generally caused by alteration in its metabolism. Overexpression of Thymidylate Synthase, for example, was mainly associated with 5-FU resistance in colorectal cancer^10^. Microarray analyses have shown that non-coding microRNAs (miRNAs) may enhance 5-FU resistance by regulating 5-FU-metabolizing enzymes^11^. The miR-433, miR-203, miR-192 and miR-215 regulate post-transcriptional expression of TS and modulate 5-FU chemosensitivity in colon cancer cells. Dihydropyrimidine dehydrogenase (DPD), the initial enzyme of 5-FU catabolism, can also be regulated by some miRNAs, including miR-27a, miR-27b, miR-582-5p, and miR-134^11^. Moreover, other mechanisms were implicated in conferring drug resistance to colorectal cancer cells such as the protection from apoptosis through the inhibition of pro-apoptotic and/or overexpression of survival proteins. Perturbation of cell cycle, preventing incorporation of 5-FU metabolites, and adaptive response to Reactive oxygen species (ROS) production have been also reported to cause 5-FU resistance^6,12^. Overexpression of ATP-binding cassette (ABC) transporters proteins including ATP-binding cassette sub-family G member 2 (ABCG2) and multidrug resistance-associated protein 1 (MDR1), known to mediate cellular efflux of the cytotoxic metabolite of 5-FU on cell membrane, is one of the key molecular mechanisms resulting in chemotherapeutic resistance^13^.

In colon cancer cells, the acquisition of invasive behavior was also related to Epithelial-mesenchymal transition (EMT) as a mechanism for 5-FU chemotherapy resistance^14^. Recent studies highlighted that overexpression of ABC transporters may be caused by the EMT as an important biological process that promotes drug resistance and tumor dissemination through deregulated expression of EMT mediators^15^.

Consequently, development of alternate strategies to improve the effectiveness of 5-FU chemotherapy and to overcome drug resistance are critically required^16^. Several studies have clearly shown that dietary polyphenols are among the naturally occurring substances that have shown promising anti-cancer properties and low toxicity in comparison to standard chemotherapeutic agents. Phenolic compounds exhibited anti-tumorigenic activities in multiple carcinogenesis pathways including the inhibition of cell proliferation, induction of apoptosis, modulation of oxidative stress, blockade of pro-inflammatory cascades and pathological angiogenesis and stimulation of anti-tumoral immune responses, which finally resulted in the arrest of cancer progression and metastasis^17,18^. An increase in the efficacy of chemotherapy and prevention of multidrug resistance are among other important effects of dietary polyphenols^19^. These compounds can not only kill cancer cells but also restore drug sensitivity^20^. Therefore, patients with colorectal cancer often adopt natural antioxidants or dietary supplements in their regimen as adjuncts to the conventional chemotherapy based on the belief that they would exhibit beneficial effects^21^. In fact, it has been shown that a combination of selected natural compounds improves the treatment efficacy of chemotherapy and increases the drug sensitivity in cancer cells^22^.

We have previously reported that peel polyphenolic extract (Peph) from the Tunisian quince (*Cydonia oblonga* Miller) displays a potent anti-tumoral effect in human colon adenocarcinoma LS174 cells. In the present study, we extend this work to investigate the anti-proliferative potentiality of each phenolic compound from total Peph extract on an in-house generated 5-FU-resistant cell line (LS174-R), in comparison with the parental 5-FU-sensitive LS174 cells. Interestingly, we found that Kaempferol, one of the phenolic compounds, can be proposed as a potential chemotherapeutic agent by its own and/or in combination to improve the sensitivity of 5-FU-resistant colon cancer cells.

## Results

### The 5-FU-resistant LS174-R cells display different characteristics from the parental one

**Figure 1:**
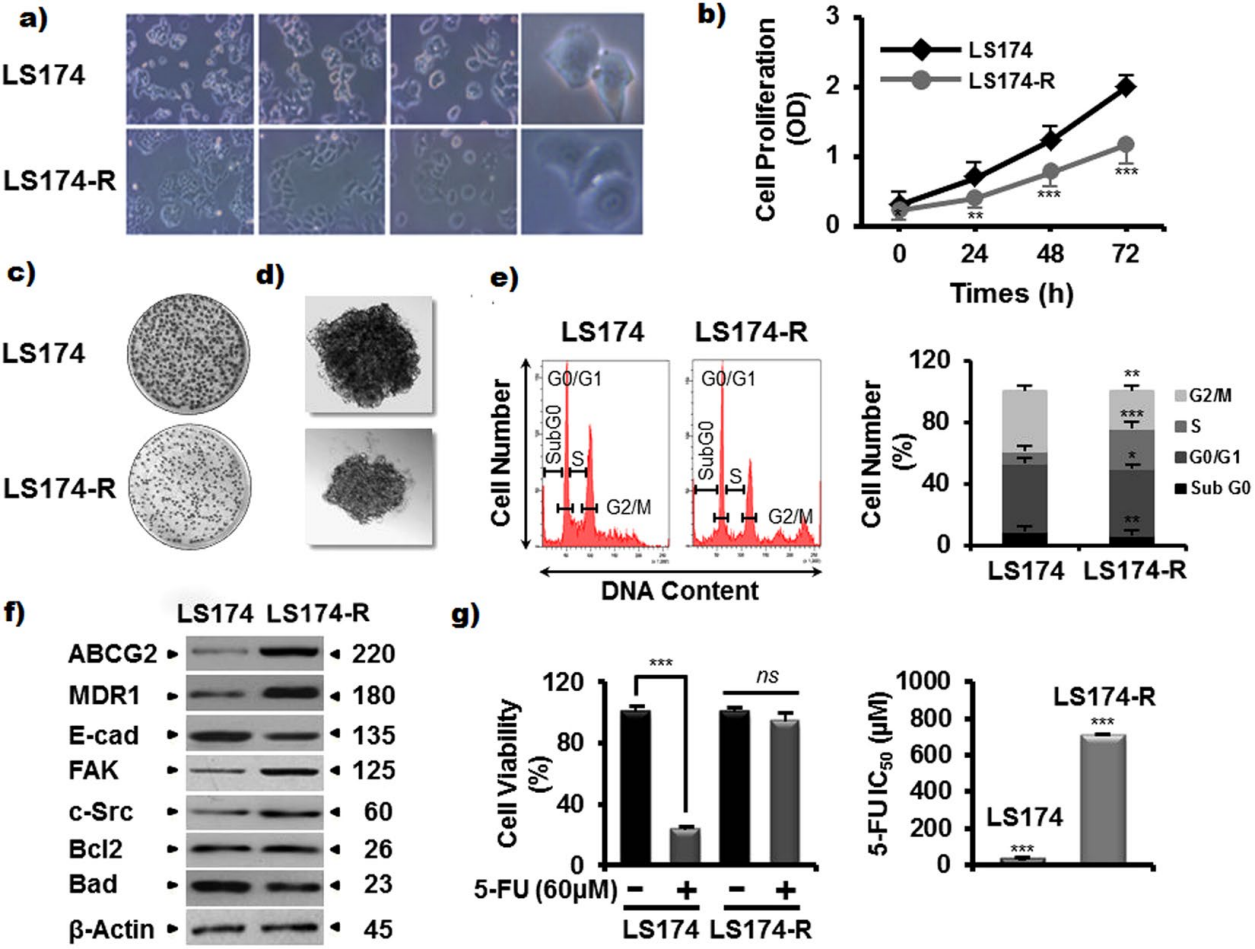
Characteristics of 5-FU-resistant LS174-R cells in comparison to the parental ones. (**a**) Representative microscopic images of both sensitive and 5-FU resistant cancer cells photographed under phase contrast microscope. (**b**) Proliferation rate of parental and 5-FU resistant cells for 24 h, 48 h and 72 h was determined by MTT assay. (**c**) Colony-forming capacity of parental and 5-FU-resistant cells was measured using clonogenic survival assay. 2000 viable cells from each group were cultured in six-well plates for additional 10 days. Colonies were stained with crystal violet and each assay was photographed. (**d**) Representative microscopy images of colon cancer cells in 3D cultures (x10). Both colon cancer cells were analyzed for spheroid formation capacity in ultra-low attachment (ULA) round bottom 96-well plates coated with agarose. (**e**) Cell cycle analysis of 5-FU-resistant cells compared to parental cells by flow cytometry using propidium iodide assay. (**f**) Protein expression of different cellular effectors was analyzed in both parental LS174 and 5-FU resistant LS174-R cells by western blot using specific antibodies. β-actin was used as a reference protein for equal loading. (**g**) Sensitive LS174 and resistant LS174-R cells were treated with different concentrations of 5-FU. The IC_50_ (50% inhibitory concentration) values were calculated at 72 h time post treatment with MTT assay. Results were normalized to each control in percentage and represented as mean ± SE of three independent experiments, each performed at least in triplicate. *p < 0.05, **p < 0.01, ***p < 0.005 and *ns: non significant*.

To mimic development of 5-Fluorouracil-resistant colorectal tumors, a 5-FU-resistant LS174-R cells were generated in-house by continuous exposure of parental sensitive cells to increasing concentrations of 5-FU (10–100 μM). Refractive cells to 60 μM of 5-Fluorouracil were obtained after 8 months of treatment and were characterized morphologically by the acquisition of an appearance different from the parental LS174 cells (Fig. 1a). They became elongated and asteroid shaped compared to the parental cell type.

For comparative study, the proliferation rate of parental LS174 cells and chemo-resistant cancer LS174-R cells was measured, over 3 days, using MTT cell viability assay. Our results showed that 5-FU-resistant cells grow slowly compared to the parental LS174 cells (Fig. 1b). Accordingly, the sensitive cells were able to produce higher number of colonies (463 colonies vs 318 for 5-FU-resistant LS174-R cells) (Fig. 1c) and larger spheroids in comparison to resistant cells which produced grape-like spheroids with small, round, single cells at the border of the compact spheres (Fig. 1d).

On the basis of the morphologic changes observed in LS174-R cells, we explored the characteristics and the molecular mechanisms driving the changes in resistant cells by analyzing the cell cycle distribution and investigating the levels of some proteins linked to 5-FU resistance such as ABC transporters, EMT protein markers and apoptosis effectors in both parental and resistant cells. As shown in Fig. 1e, the proportion of LS174-R cells (26.5%) in the S phase was higher than that of the parental LS174 cells (7.5%) and decreased in the G2/M phase (20.7% vs 34.7% in parental cells).

Western blot analysis indicated that chemo-resistant LS174-R cells expressed high levels of MDR1, ABCG2, c-Src and FAK proteins along with reduced expression of Bad and E-cadherin proteins relative to parental LS174 cells. Bcl2 expression was similar in both cell lines (Fig. 1f). However, we could not detect vimentin and Bax proteins in both sensitive and resistant cells, which is in accordance with previous work reporting that LS174 cells lack expression of these two proteins^23,24^.

To highlight the adaptation and acquired resistance of refractive cells to high concentrations of 5-Fluorouracil, the viability of parental and resistant cells at different doses of the drug was investigated using MTT assay. As shown in Fig. 1g, the IC_50_ value of 5-FU in resistant LS174-R cells (IC_50_ = 706 µM) was 26-fold higher than that of sensitive LS174 cells in which 26.9 µM were needed to kill 50% of the parental cells (Fig. 1g).

### Kaempferol inhibits the viability of 5-FU-resistant LS174-R cancer cells

**Figure 2:**
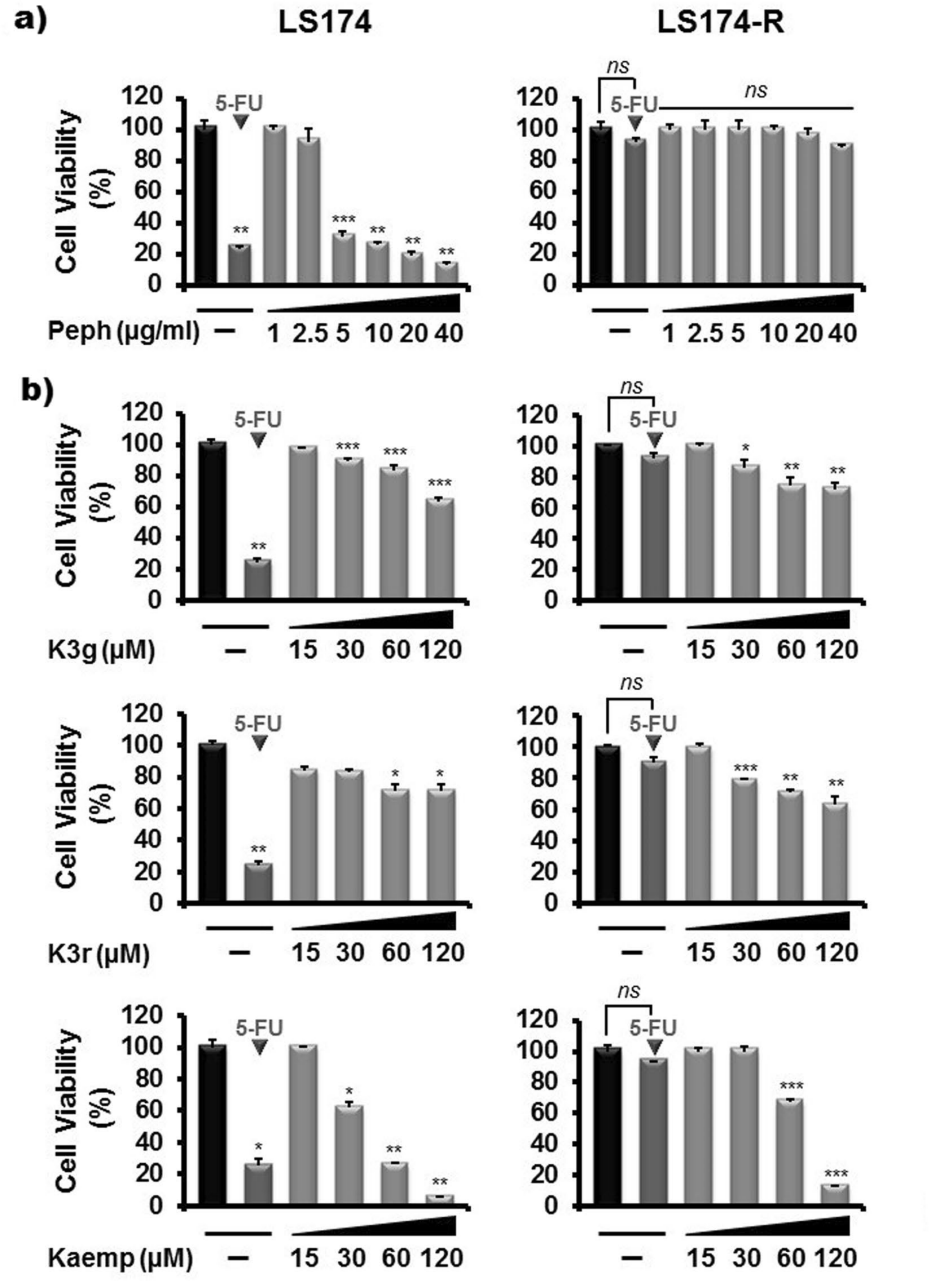
Kaempferol overcomes 5-FU resistance in LS174-R cancer cells. Parental LS174 cells and resistant LS174-R cells were cultured in 96-well plates and treated with increasing concentrations of (**a**) *Cydonia oblonga* Miller peel polyphenolic extract (Peph) (1–40 µg/ml) and (**b**) Kaempferol and its analogs, Kaempferol 3-O-glucoside (K3g) and Kaempferol 3-O-rutinoside (K3r), (15–120 µM) for 72 h. Cell viability was measured by MTT assay. The absorbance was measured at 540 nm. Results were normalized to each control in percentage and represented as mean ± SE of three independent experiments. *p < 0.05, **p < 0.01, ***p < 0.005 when compared to their respective CN and *ns: non significant*.

To assess the effect of quince peel polyphenolic extract on growth of 5-FU-resistant LS174-R cancer cells, MTT assay was employed. For comparative study, both parental and chemo-resistant cancer cells were treated for 72 h with increasing concentrations (0–40 µg/ml) of Peph polyphenolic extract. Consistent with our previous findings^25^, Peph significantly reduced the viability of parental LS174 cells in a dose-dependent manner. However, no significant inhibitory effect was observed in the growth of 5-FU-resistant LS174-R cells (Fig. 2a). Thereby, we proposed to evaluate the effect of each phenolic compound identified in the total peel polyphenolic extract on the viability of chemo-resistant LS174-R cells compared to parental LS174 cells. As summarized in Tables 1 and 2, phenolic compounds tested at equivalent concentrations to that present in 5 µg/ml, 10 µg/ml and 20 µg/ml of the total peel polyphenolic extract failed to induce any significant effect on cell viability in both parental and resistant cells.

**Table 1:** Effect of quince peel compounds on the viability of sensitive LS174 cells.

Treatment (µM)	Cell Viability (%)						
Mock	100 ± 0.3						
5-FU (60 µM)	25 ± 0.9						
Compounds	% 5 µg/ml	% 10 µg/ml	% 20 µg/ml	15 µM	30 µM	60 µM	120 µM
**Q**	100 ± 1.2	97 ± 0.2	96 ± 0.6	63 ± 1.3	41 ± 0.3	33 ± 0.7	20 ± 0.2
**R**	90 ± 0.5	50 ± 0.9	44 ± 0.9	41 ± 1.1	42 ± 0.23	38 ± 0.3	31 ± 1.3
**+C**	100 ± 0.43	100 ± 0.32	97 ± 0.81	98 ± 0.54	41 ± 1.63	40 ± 0.52	37 ± 0.5
**−C**	100 ± 0.63	91 ± 0.3	72 ± 0.63	56 ± 0.44	48 ± 0.61	47 ± 0.42	38 ± 0.21
**H**	100 ± 0.51	100 ± 0.91	99 ± 0.56	84 ± 1.4	38 ± 1.3	37 ± 1.63	35 ± 1.7
**I**	100 ± 0.3	100 ± 0.53	91 ± 0.57	50 ± 0.64	48 ± 0.81	47 ± 0.29	39 ± 0.77
**ChA**	93 ± 0.48	91 ± 0.6	90 ± 0.72	84 ± 0.7	85 ± 0.66	84 ± 0.35	41 ± 0.58
**CrA**	100 ± 1.6	100 ± 0.33	100 ± 0.9	89 ± 1.6	51 ± 1.6	30 ± 1.4	25 ± 0.93
**NeA**	100 ± 0.77	100 ± 0.63	90 ± 0.42	80 ± 0.3	46 ± 0.23	38 ± 0.53	34 ± 0.74
**PcA**	100 ± 0.73	98 ± 1.3	94 ± 1.2	64 ± 0.99	58 ± 0.92	48 ± 0.71	32 ± 0.81
**K3g**	100 ± 0.33	100 ± 0.51	100 ± 0.14	97 ± 0.26	**89** ± 0.4	**83** ± 0.21	**63** ± 1.1
**K3r**	100 ± 0.66	100 ± 0.3	100 ± 0.42	84 ± 0.73	**83** ± 0.3	**79** ± 1.1	**71** ± 1.3
**Kaemp**	100 ± 0.13	100 ± 0.23	100 ± 0.76	100 ± 0.16	**62** ± 0.83	**26** ± 0.91	**5** ± 0.13

Each phenolic compounds [Quercetin (Q), Rutin (R), (+)-Catechin (+C), (−)-Catechin (−C), Hyperin (H), Isoquercitrin (I), Chlorogenic acid (ChA), Cryptochlorogenic acid (CrA), Neochlorogenic acid (NeA), p-coumaric acid (PcA), Kaempferol-3-O-glucoside (K3g), Kaempferol-3-O-rutinoside (K3r) and Kaempferol (Kaemp)] was tested at equivalent concentrations to that present in 5 µg/ml, 10 µg/ml and 20 µg/ml of the total peel polyphenolic extract and at increasing concentrations (15–120 µM). Cell viability was determined by MTT assay after 72 h of treatment. The absorbance was measured at 540 nm. Results were normalized to each control in percentage and represented as mean ± SE of three independent experiments.

**Table 2:** Effect of quince peel compounds on the viability of resistant LS174-R cells.

Treatment (µM)	Cell Viability (%)						
Mock	100 ± 0.61						
5-FU (60 µM)	93 ± 0.17						
Compounds	% 5 µg/ml	% 10 µg/ml	% 20 µg/ml	15 µM	30 µM	60 µM	120 µM
**Q**	100 ± 0.12	100 ± 0.71	100 ± 0.3	100 ± 0.9	100 ± 1	100 ± 0.7	100 ± 0.4
**R**	100 ± 0.21	100 ± 0.12	100 ± 0.22	100 ± 0.29	98 ± 0.38	87 ± 0.49	86 ± 0.6
**+C**	100 ± 0.63	100 ± 0.24	100 ± 0.34	100 ± 0.5	100 ± 1	100 ± 1.3	98 ± 0.39
**−C**	100 ± 1.4	100 ± 0.7	100 ± 0.98	100 ± 0.13	98 ± 0.77	91 ± 0.52	84 ± 0.31
**H**	100 ± 0.43	100 ± 0.18	100 ± 0.22	100 ± 0.43	100 ± 0.48	94 ± 0.18	91 ± 0.7
**I**	100 ± 0.41	100 ± 0.45	100 ± 0.22	100 ± 0.7	100 ± 0.59	93 ± 0.19	90 ± 0.46
**ChA**	100 ± 0.26	100 ± 0.48	100 ± 0.49	100 ± 0.8	100 ± 0.32	100 ± 0.22	100 ± 0.63
**CrA**	100 ± 0.48	100 ± 0.46	100 ± 0.84	100 ± 0.7	100 ± 0.53	100 ± 0.3	82 ± 0.23
**NeA**	100 ± 0.7	100 ± 0.12	100 ± 0.3	100 ± 0.26	91 ± 0.14	92 ± 0.37	82 ± 0.12
**PcA**	100 ± 1	100 ± 0.45	100 ± 1.01	100 ± 0.7	99 ± 1.06	98 ± 0.38	94 ± 0.7
**K3g**	100 ± 0.2	100 ± 0.46	100 ± 0.2	100 ± 0.54	**86** ± 0.6	**74** ± 0.13	**70** ± 0.32
**K3r**	100 ± 0.53	100 ± 0.32	100 ± 0.59	100 ± 0.4	**79** ± 0.6	**71** ± 0.59	**63** ± 0.13
**Kaemp**	100 ± 0.21	100 ± 0.49	100 ± 0.56	100 ± 0.12	100 ± 0.24	**67** ± 0.17	**13** ± 0.56

Each phenolic compounds [Quercetin (Q), Rutin (R), (+)-Catechin (+C), (−)-Catechin (−C), Hyperin (H), Isoquercitrin (I), Chlorogenic acid (ChA), Cryptochlorogenic acid (CrA), Neochlorogenic acid (NeA), p-coumaric acid (PcA), Kaempferol-3-O-glucoside (K3g), Kaempferol-3-O-rutinoside (K3r) and Kaempferol (Kaemp)] was tested at equivalent concentrations to that present in 5 µg/ml, 10 µg/ml and 20 µg/ml of the total peel polyphenolic extract and at increasing concentrations (15–120 µM) after 72 h of treatment. Cell viability was determined by MTT assay. The absorbance was measured at 540 nm. Results were normalized to each control in percentage and represented as mean ± SE of three independent experiments.

Interestingly, we found that increasing concentrations (0–120 µM) of each phenolic compound inhibit the growth of sensitive LS174 cells in a dose-dependent manner after 72 h of treatment. However, only Kaempferol and its analogs (Kaempferol 3-O-glucoside and Kaempferol 3-O-rutinoside) were able to reduce the viability of 5-FU-resistant cells (Table 2 and Fig. 2b). As shown in Fig. 2b, at a concentration of 120 µM, Kaempferol induced the most effective inhibitory effect with more than 80% of growth inhibition while this effect did not exceed 30% and 37% for Keampferol 3-O-glucoside (K3g) and Kaempferol 3-O-rutinoside (K3r), respectively, after 72 h of treatment.

### Kaempferol chemo-sensitizes resistant cancer cells to 5-FU chemotherapy

For comparative chemosensitivity study, the parental and 5-FU-resistant cells were analyzed by MTT and colony formation assays for cellular growth following treatment with 5-FU alone or combined with increasing concentrations (0–120 µM) of Kaempferol. Both colon cancer cells were pretreated for different time (8 h, 12 h and 24 h) with Kaempferol (1–75 µM) and then exposed to 60 µM of 5-FU after removing or preserving the phenolic compound in the culture medium for additional 72 h. We found that Kaempferol pretreatment decreased the growth of parental and 5-FU-resistant cancer cells in dose and time-dependent manners (see Supplementary Fig. S1). Kaempferol, at 75 µM concentration, was able to induce 40% of growth inhibition in 5-FU-resistant LS174-R cells after 24 h of pretreatment. This effect was improved to about 70% when Kaempferol was not removed from the culture medium (see Supplementary Fig. S1).

**Figure 3:**
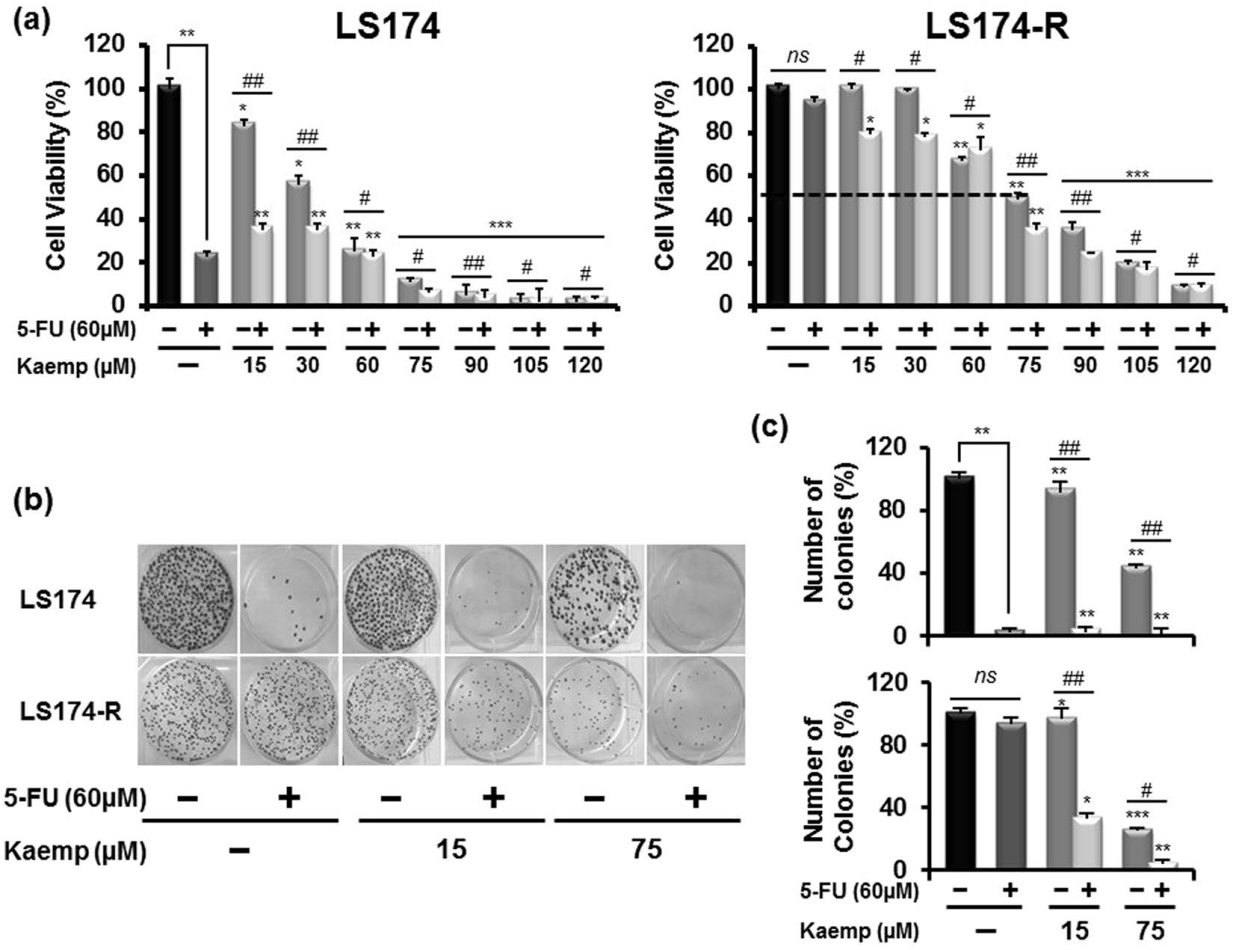
Kaempferol chemo-sensitizes resistant colon cancer cells to 5-FU chemotherapy. (**a**) Parental LS174 cells and resistant LS174-R cells were cultured in 96-well plates and treated with a concentration range from 15 µM to 120 µM of Kaempferol alone, 5-FU (60 µM) alone and the combination of both for 72 h. Cell viability was measured by MTT assay. The absorbance was measured at 540 nm. Assays were performed in triplicate. (**b**) Kaempferol effect on parental LS174 cells and resistant LS174-R cells colony-forming capacity was measured using clonogenic survival assay. Colon cancer cells were treated with vehicle (control) or Kaempferol (15 and 75 µM) combined or not to 60 µM of 5-FU for 72 h. After removal of the medium, 2000 viable cells from each group were cultured in six-well plates for additional 10 days. Colonies were stained with crystal violet and each assay was photographed. (**c**) The number of colonies was analyzed and scored by CFU scope quantification software. Results are expressed as the number of colony forming cells per well in percentage and normalized to control (vehicle, considered to represent 100%). Graphs are represented as mean ± SD of three independent experiments. *p < 0.05, **p < 0.01 and ***p < 0.005 when compared to their respective controls. ^*#*^*p* < 0.05, ^*##*^*p* < 0.01 (Kaempferol + 5-FU groups vs Keampferol groups).

Interestingly, when cells were treated with Kaempferol at concentrations exceeding 75 µM (15–120 µM) for 72 h, this phenolic compound significantly decreased cell proliferation of both parental and chemo-resistant cells in a concentration-dependent manner. The IC_50_ of Kaempferol was approximately 75 µM on LS174-R cells after 72 h of exposure. This inhibitory effect was enhanced to about 65% after the combined treatment with 60 µM of 5-FU (Fig. 3a). It should be noted that 5-FU alone did not affect the viability of LS174-R cells at 60 µM concentration (Fig. 3a).

Thus, on the basis of this result, the Kaempferol’s concentration of 75 µM was selected for further investigations. Our data showed that Kaempferol notably sensitized the chemo-resistant LS174-R cells to 5-FU treatment starting from low dose (15 µM) (Fig. 3), which suggests a synergistic or additive anti-tumor effect between 5-FU and Kaempferol in both 5-FU-sensitive and resistant cells.

### Pharmacological interaction between 5-Fluorouracil and Kaempferol

Because certain drug interactions can lead to a loss of therapeutic efficacy^26^, a combination studies between Kaempferol and 5-Fluorouracil were performed on the viability of 5-FU-resistant LS174-R and parental LS174 cells. Our result showed that combined treatment of 5-FU with Kaempferol reduced the 5-FU-IC_50_ values in parental LS174 cells (from 29.9 to 20.9 µM) and 5-FU-resistant LS174-R cells (from 706 to 274.2 µM). Interestingly, the analysis of drug interaction after calculating the Combination Index (CI) revealed an additive effect (CI = 1) in sensitive cells and confirmed a synergism (CI < 1) in 5-FU-resistant cells (Table 3).

**Table 3:** Drug and compound interaction in parental and 5-FU-resistant LS174 colon cancer cells.

Cell line	Treatment	IC_50_ (µM)	Combination Index (CI)
LS174	Kaempferol	44.76 ± 1.06	1
	5-FU	26.9 ± 0.36	
LS174-R	Kaempferol	72.98 ± 0.19	0.6
	5-FU	706 ± 0.28	

Combination index values for Kaempferol and 5-Fluorouracil was calculated after 72 h of treatment of sensitive LS174 and 5-FU-resistant LS174-R cells at 50% growth inhibition.

CI <1 indicates synergism, CI = 1 additive effect and CI >1 antagonism.

### Kaempferol induces cell cycle arrest of colon cancer cells

**Figure 4:**
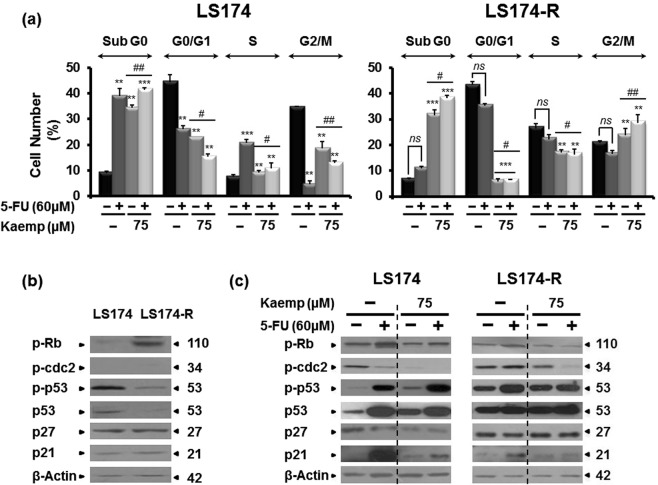
Kaempferol induces cell cycle arrest of sensitive and 5-FU-resistant cancer cells. (**a**) Cell cycle phase distribution of cancer cells cultured in the absence (mock) and the presence of Kaempferol (75 µM) alone or combined to 60 µM of 5-FU for 72 h were analyzed by flow cytometry using propidium iodide assay. Results were represented as mean ± SE of three independent experiments. *p < *0.05*, **p < *0.01*, ***p < *0.005* when compared to their respective CN, ^*#*^*p* < 0.05, ^*##*^*p* < 0.01 (Kaempferol + 5-FU groups vs Keampferol groups) and *ns: non significant*. Cell cycle-related proteins were analyzed by western blotting in (**b**) 5-FU resistant cells versus parental cells and (**c**) after cell treatments with Kaempferol, 5-FU, and the combination of both for 72 h. β-actin was used as a loading control. The data shown are representative of three independent experiments.

Cell cycle perturbation can be involved in acquired 5-FU resistance^27^. In accordance with this, we analyzed the cell cycle phase distribution of both resistant and parental cancer cells after 72 h of treatment with Kaempferol alone or combined with 60 µM of 5-Fluorouracil. As shown in Fig. 4a, 5-FU treatment induced accumulation of sensitive LS174 cells in the S phase of the cell cycle (from 7.5 to 20.6%) along with a decrease of cells in the G2/M phase (from 34.7 to 4.6%), compared to the mock-treated cells. However, no significant effect was observed on refractive LS174-R cells to 60 µM of 5-FU. Kaempferol at 75 µM alone or in combination with 5-FU increased parental cells proportion in the S phase (from 7.5 to 9.2% and to 10.7% respectively) and induced a decrease in the G2/M phase (from 34.7 to 18.5% and to 12.9% respectively). However, the resistant LS174-R cells were accumulated in G2/M phase only after the concomitant use of Kaempferol with 60 µM of 5-FU (from 20.7 to 28.7%). This effect was associated with an increase in sub-G0 population (more than 30%) in both sensitive and 5-FU resistant cancer cells (Fig. 4a).

**Figure 5:**
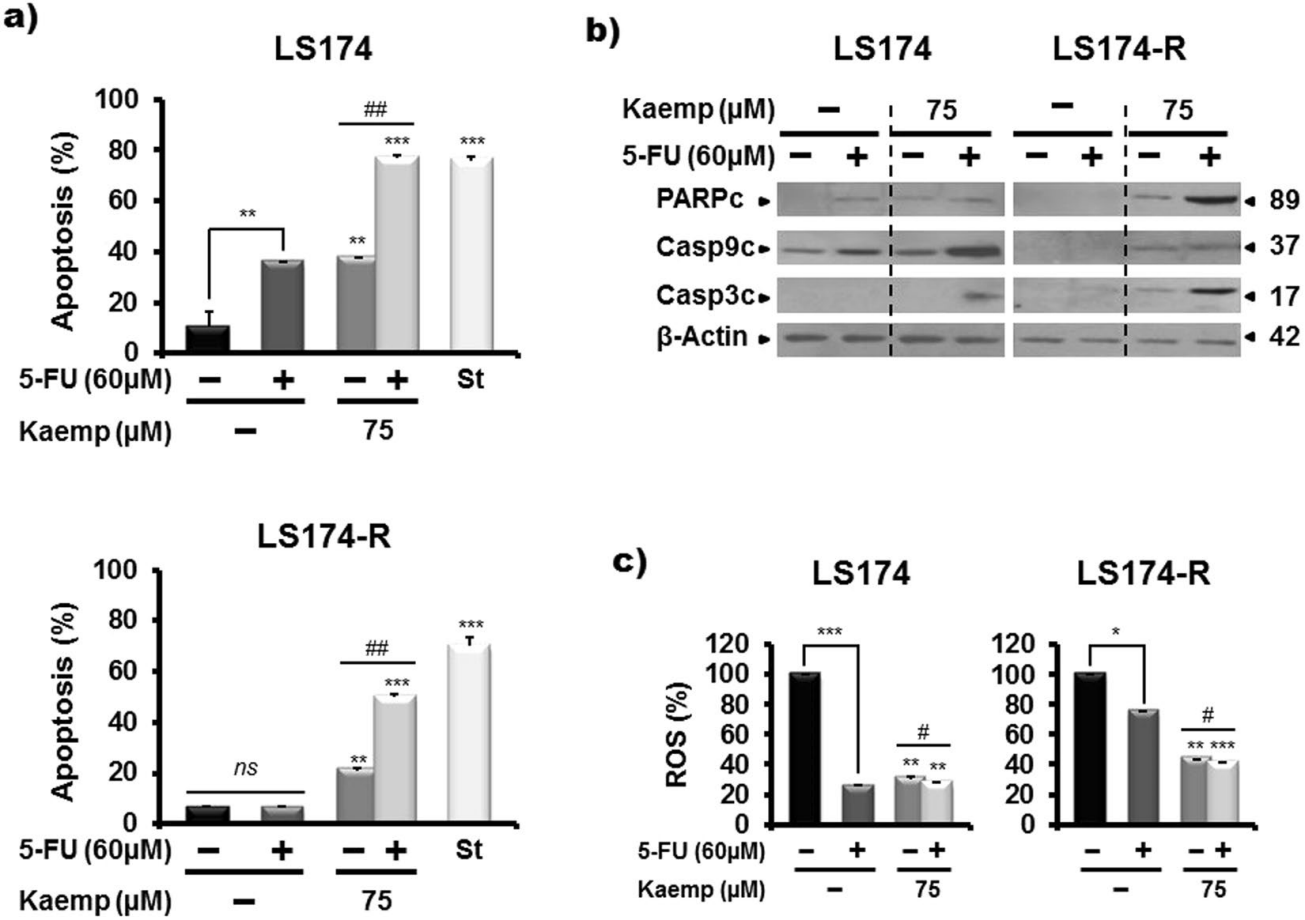
Kaempferol chemosensitizes 5-FU-resistant cancer cells to apoptosis and reduces the ROS production. (**a**) Apoptosis detection in mock and treated-cells with Kaempferol alone or combined to 5-FU for 72 h by flow cytometry analysis using annexin-V/7-AAD staining. Staurosporin (2 µM, St) was used as a positive control of apoptosis. Results were represented as mean ± SE of three independent experiments. *p < 0.05, **p < 0.01, ***p < 0.005 when compared to their respective CN, ^*#*^*p* < 0.05, ^*##*^*p* < 0.01 (Kaempferol + 5-FU groups vs Keampferol groups) and *ns: non significant*. (**b**) Western blot analysis of some apoptosis-related proteins in both parental LS174 and 5-FU resistant LS174-R cells after 72 h of treatment with Kaempferol, 5-FU and the combination of both. β-actin was used as a reference protein for equal loading. The data shown are representative of three independent experiments. (**c**) Histograms analysis of ROS production measured with CMH2DCFDA staining after 72 h of treatment with Kaempefrol, 5-FU and the combination of both in parental LS174 and 5-FU-resistant LS174-R cells. Detection of ROS was related to the quantity of subsequent oxidation leading emitting fluorescence. Data are reported as the means ± S.E.M of three independent experiments. *p < 0.05, **p < 0.01, *** p < 0.005 with respect to mock-treated controls; ^*#*^*p* < 0.05, ^*##*^*p* < 0.01 (Kaempferol + 5-FU groups vs Keampferol groups).

To further characterize the mechanism by which Kaempferol induced cell growth arrest, we investigated the levels of some proteins involved in cell cycle arrest in both parental and 5-FU-resistant cancer cells. As shown in Fig. 4b, p21, p27 and cdc2 proteins were expressed at the same levels in both sensitive and chemo-resistant cells. However, chemo-resistant LS174-R cells expressed high level of the phosphorylated form of retinoblastoma (Rb), compared to parental LS174 cells where the level of phospho-p53 protein was increased. Kaempferol treatment of sensitive LS174 cells decreased the expression of p27 and the phospho-Rb, cdc2 and enhanced p53 and phospho-p53 (at Ser15) expression. The combined treatment (Kaempferol and 5-Fluorouracil) induced an increase in the expression level of p21, p53 and its phosphorylated form (at Ser15) along with a decrease in phospho-cdc2 and p27 expression levels (Fig. 4c). No effect was observed on the expression of phospho-Rb protein. Interestingly, while Kaempferol slightly decreased phospho-cdc2 and phospho-Rb proteins along with induced expression of phospho-p53 (at Ser15) and p21 in LS174-R cells, the combined treatment-mediated arrest of resistant cells in the G2-M phase was associated with the loss of the phosphorylated forms of Rb and cdc2 without any effect on the levels of p21 and p27 proteins (Fig. 4c).

### Kaempferol induces a caspase-dependent apoptosis of resistant cancer cells

Resistance to apoptosis is one of the most important features of cancer^28^. The increase of sub-G0 population after Kaempferol treatment suggests that growth inhibition of colon cancer cells may be explained by cell apoptosis. Flow cytometric analysis (Fig. 5a) showed that, as expected, 5-FU treatment had no apoptotic effect on chemo-resistant LS174-R cells while 39% of the sensitive LS174-treated cells underwent apoptosis. Interestingly, the combined treatment with 5-FU and 75 µM Kaempferol increased the percentage of apoptotic cells to 76.8% in sensitive LS174 cells and to 50% in chemo-resistant LS174-R cells (Fig. 5a). The pro-apoptotic effect was associated with the activation of caspase 3 and caspase 9 and cleavage of PARP (Fig. 5b), suggesting that Kaempferol treatment chemo-sensitized resistant cancer cells to apoptosis through caspases-dependent mechanisms.

### Antioxidant activity of Kaempferol in colon cancer cells

It has been reported that Kaempferol reduces the cancer risk by increasing the body’s antioxidant defense against free radicals^29^. We thus assessed the effect of Kaempferol alone or combined with 5-FU on the intracellular redox status of parental LS174 cells and 5-FU-resistant cells. Our finding showed that Kaempferol at 75 μM reduced the reactive oxygen species (ROS) production by 69% and 56% after 72 h of treatment, in the sensitive and resistant cells, respectively (Fig. 5c). Combined treatment increased the inhibitory effect of Kaempferol on ROS production in both sensitive (to 73%) and chemo-resistant cells (to 60%).

### Kaempferol blocks survival signaling pathways in colon cancer cells

**Figure 6:**
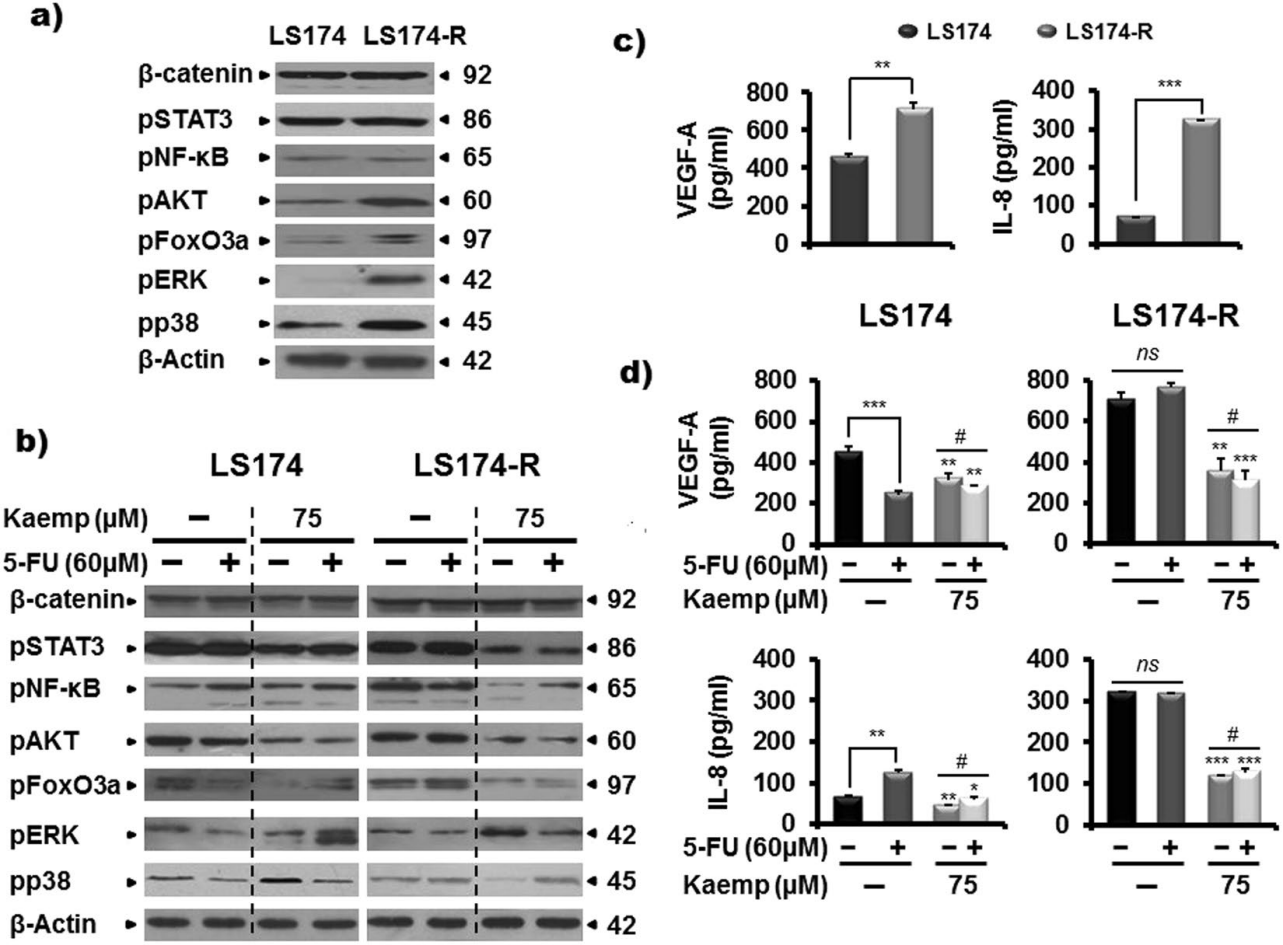
Kaempferol modulates survival signaling pathways in both sensitive and resistant colon cancer cells and reduces the production of the two angiogenic factors, VEGF-A and IL-8 in 5-FU-refractive LS174-R cells. (**a**) Different cellular effectors were monitored in chemo-resistant LS174-R cells and sensitive LS174 cells by western blot using specific antibodies. β-actin was used as a loading control. (**b**) 5-FU-sensitive and resistant cancer cells were treated with Kaempferol, 5-FU, and the combination of both for 72 h. Protein extracts (30 µg) from whole cell lysates were analysed by western blotting using specific antibodies. β-actin was used as a reference protein for equal loading. One representative experiment of three independent ones was shown. (**c**) VEGF and IL-8 secretion were determined by human VEGF and IL-8 ELISA Kit in both control sensitive and 5-FU-resistant LS174-R cells. (**d**) Supernatants from parental LS174 and 5-FU resistant LS174-R cells cultured in the absence (vehicle) or presence of Kaempferol (75 µM) combined or not to 60 µM of 5-FU were collected and analyzed by Human VEGF and IL-8 specific ELISA. Results are reported as the mean ± SE of three independent experiments each run in triplicate (*p < 0.05, **p < 0.01, *** p < 0.005*;* ^*#*^*p* < 0.05, ^*##*^*p* < 0.01 (Kaempferol + 5-FU groups vs Keampferol groups and *ns: non significant*). The data were corrected to the cell number.

To further characterize the mechanisms by which Kaempferol chemosensitizes the resistant colon cancer cells to 5-FU treatment, we analyzed the expression level of some proteins involved in JAK/STAT3, Wnt/β-catenin, mitogen activated protein kinases (MAPK) cascade, phosphatidyl-3-phosphate kinase (PI3K)/AKT and NF-κB signaling pathways that play critical role in the development and progression of colorectal cancer^30^. Western blot analysis showed increased phosphorylation of extracellular-regulated kinases 1/2 (ERK1/2), p38 MAPK and AKT in chemo-resistant LS174-R cells compared to sensitive LS174 cells (Fig. 6a), suggesting the acquisition of more aggressive cell features related to the activation of MAPK and PI3K/AKT signaling pathways.

After 72 h of treatment, 5-FU reduced the activation of ERK1/2 and p38 in sensitive tumor cells without any noticeable effect in chemo-resistant LS174-R cells. Interestingly, Kaempefrol alone or combined with 5-FU modulated the phosphorylation levels of the tested proteins in both parental and chemo-resistant cancer cells. As shown in Fig. 6b, Kaempferol treatment increased the expression of phospho-ERK1/2 associated to the inhibition of phospho-p38 kinase in resistant LS174-R cell line. The same effect was detected in sensitive LS174 cells with the combined treatment. This data suggests the existence of a cross-talk between ERK and p38 activation^31^.

Moreover, Kaempferol alone or combined with 5-FU reduced the phosphorylated form of STAT3 and the one of the pro-survival kinase AKT and its target tumor suppressor FOXO3a transcription factor, in both sensitive and resistant tumor cells (Fig. 6b). Our result also supports the inhibition of NF-κB in the refractive cancer cell line model, whereas it was slightly activated in treated-parental LS174 cells with the Kaempferol combined to 5-FU. However, no effect was detected on β-catenin activity for both parental and resistant cancer cells (Fig. 6b).

### Kaempferol inhibits the production of VEGF-A and IL-8 angiogenic factors

Tumor growth depends in angiogenesis^32^. Therefore, we assessed the production of vascular endothelial growth factor (VEGF) and interleukin-8 (IL-8) known as the major positive regulators of angiogenesis. ELISA analysis highlighted that the secretion of IL-8 by chemo-resistant LS174-R cells was five times higher than that of LS174 sensitive cells. Similarly, LS174-R cells produced VEGF-A (707 pg/ml) to a greater extent than the parental LS174 cells (450 pg/ml) (Fig. 6c). This finding supports that LS174-R cells acquired other malignant characteristics than parental cells through expressing high amounts of IL-8 and VEGF-A proteins.

The treatment of sensitive cells with 5-FU modulated the VEGF-A and IL-8 cytokines secretion without affecting their levels in chemo-resistant tumor cells. Interestingly, Kaempferol alone or combined with 5-FU inhibited the production of the two angiogenic regulators in both colon cancer cells. ELISA analysis showed a 50% decrease of VEGF-A (from 707 to 355 pg/ml) and IL-8 (from 319 to 116 pg/ml) secretion after 72 h of treatment with Kaempefrol in chemo-resistant LS174-R cells. Interestingly, the combined treatment reduced also the production of VEGF-A (from 707 to 308 pg/ml) and IL-8 (from 319 to 127 pg/ml) (Fig. 6d) in 5-FU-resistant cells (Fig. 6d).

### Kaempferol modulates the expression levels of enzymes involved in 5-FU metabolism

**Figure 7:**
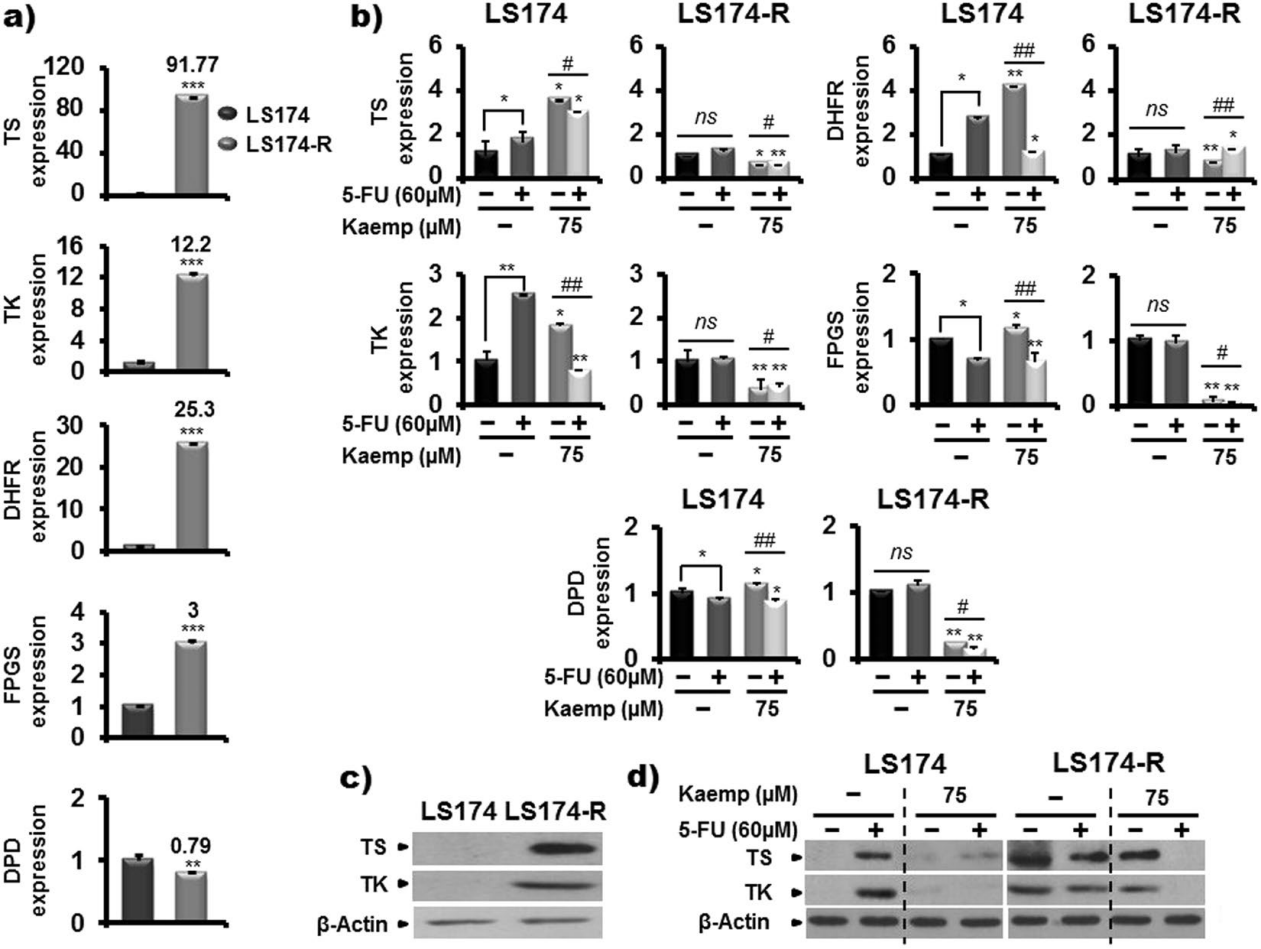
Kaempferol modulates the expression levels of enzymes involved in 5-FU metabolism. (**a**) The amounts of mRNA transcripts of five genes, Thymidylate synthase (TS), Thymidine kinase (TK), Dihydrofolate reductase (DHFR), Folylpolyglutamate synthetase (FPGS) and Dihydropyrimidine dehydrogenase (DPD), involved in 5-FU metabolism were quantified by real time PCR in control sensitive and 5-FU-resitant cells and (**b**) in LS174 and LS174-R cells cultured in the absence (mock) and the presence of Kaempferol (75 µM) and/or 5-FU (60 µM) for 72 h. The values are normalized to GAPDH gene and the control value was taken as 1. Results are reported as the mean ± SE of three independent experiments each run in duplicate (*p < 0.05, **p < 0.01, *** p < 0.005; ^*#*^*p* < 0.05, ^*##*^*p* < 0.01 (Kaempferol + 5-FU treatments vs Keampferol groups), *ns: non significant*) (**c**) TS and TK protein levels were assessed by western blotting in both control 5-FU-sensitive LS174 and resistant LS174-R cells and in (**d**) colon cancer cells after treatment with vehicle (mock), Kaempferol (75 µM), 5-FU (60 µM), and the combination of both for 72 h. β-actin was used as a reference protein for equal loading.

To further identify the molecular mechanism of 5-FU resistance in the generated chemo-resistant cancer cells, we investigated the expression level of five genes involved in drug metabolism such as, thymidylate synthase (TS), thymidine kinase (TK), dihydrofolate reductase (DHFR) and the folylpolyglutamate synthetase (FPGS) implicated in 5-FU anabolism and dihydropyrimidine dehydrogenase (DPD), the key enzyme of 5-FU catabolism^33^. Quantitative PCR analysis indicated an increase in mRNA levels of TS, TK, DHFR and FPGS in chemo-resistant cancer cells with a slight decrease in DPD expression compared to parental LS174 cells (Fig. 7a). Treatment of the cells with Kaempferol alone or combined with 5-FU significantly reduced the mRNA levels of the studied genes associated to 5-FU metabolism in 5-FU-resistant cells (Fig. 7b). These results were confirmed by western blotting analysis that showed a high protein levels of TS and TK in the 5-FU-resistant cells, compared to sensitive LS174 cells (Fig. 7c), while the Kaempferol alone or in combination with 60 µM of 5-FU decreased the expression of the two proteins in the resistant cells (Fig. 7d).

### MAPK14 (p38) and PIM1 are two putative targets for Kaempferol

**Figure 8:**
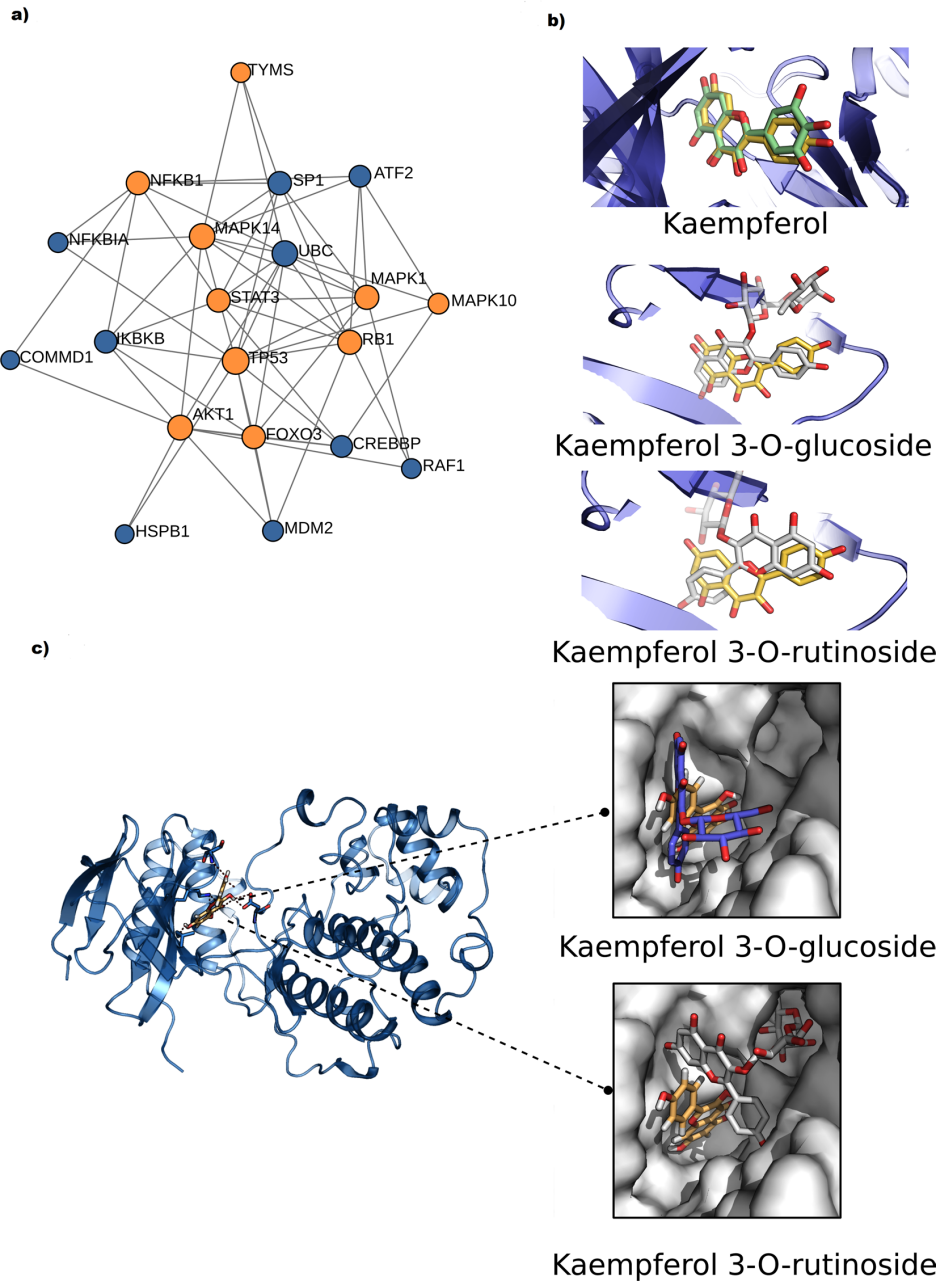
Computational interaction study of Kaempferol and its analogs with putative human cell proteins. (**a**) Interaction network of the different proteins from which expression was evaluated by the western blot assay (represented by yellow nodes). Other proteins (blue nodes) emerged from the analysis as connectors of the input proteins. The degree of centrality is proportional to the node radius. (**b**) Docking of Kaempferol (yellow sticks) with PIM1. The first panel shows the good superposition of Kaempferol with Fisetin structure (Green sticks). The second and the third panel show the docking complexes of Kaempferol 3-O-rutinoside (K3r) and Kaempferol 3-O-glucoside (K3g) with PIM1 respectively, compared to the Kaempferol. (**c**) Docking solutions of Kaempferol (Yellow sticks) with the ATP binding site in MAPK14 (p38). The two other panels show the position of Kaempferol in the interaction site in comparison to the retained complexes for K3g and K3r. Unlike Kaempferol, these two ligands cannot attain the depth of the binding pocket.

Considering the contribution of the Thymidylate synthase in acquiring the resistance to the treatment with 5-FU, we explored the putative interaction of the enzyme with Kaempferol in the cell signaling context. We established the protein-protein interaction network of 10 different signaling members, which expression levels were confirmed by western blot. The network consisted of 61 edges connecting 20 nodes, corresponding to interacting proteins. The highest betweenness scores were those for TP53, UBC and MAPK14 (p38) evaluated at 29.44, 16.3 and 16.03, respectively. Thymidylate synthase (TYMS) was situated on the edge of the network establishing connectivities with MAPK14, UBC and SP1 only with low betweenness score of 0.14 (Fig. 8a).

We then verified if any of the network nodes were previously identified as a target for Kaempferol or other members of the flavonoid family by exploring the BindingDB^34^. From the 61 entries described for the Kaempferol, MAPK14 (p38α) was identified as putative target for Kaempferol along with two other proteins, CDK6 and PIM1 which are related to RBI/MAPK14 and FOXO3/STAT3, respectively members of the INPUT protein list.

The PharMApper server also was used to predict the potential cellular targets of Kaempferol starting from its 3D structure. The PharMApper report showed that uridine cytidine kinase 2 is a potential target with the highest fit score. CDK6 was situated at the 4^th^ rank of the list, while MAPK14 (p38α) was ranked at the 30^th^ position. MAPK14 occurred six other times in the list, probably because of the redundancy in the PDB data bank. However, the Thymidylate synthase and PIM1 had no appearances in the returned list even though their structure were previously solved^35^.

### Molecular docking of Kaempferol and its analogs with their putative targets

We further investigated the putative interaction of Kaempferol with two of the identified protein targets, MAPK14 (p38) and PMI1, by proceeding with molecular docking. We also studied the interaction of the two other flavonoid components, Kaempferol-3-O-glucoside (K3g) and Kaempferol-3-O-rutinoside (K3r) (Figs 8b and S2).

Interestingly, we found that the co-crystals of four flavonoids, Myrecitin, Pentahydroxy-flavone, Quercetin and Quercetagetin with PIM1 were previously solved (PDB codes 2O3P, 2O63, 2O64, 2O65, respectively). To conduct the docking with Kaempferol, K3g and K3r, we used the bound structure of PIM1 with Myricetin (PDB code 2O63).

The spherical docking domain on PIM1 structure was centered on the binding sites of Myricetin. For MAPK14 (p38α), we proceeded according to Goettert *et al*.^36^.

The best docking scores among the retained solutions for Kaempferol against PIM1 reproduced the same interaction mode of Myricetin (Fig. 8b). For K3r, its orientation preserved the geometry of the phenyl group while the hetero-cyclic moiety was flipped in comparison to Kaempferol. For K3g, both the phenyl group and the hetero-cyclic moiety were disposed in opposing directions relative to Kaempferol. For both K3r and K3g, it seems that the glycosyl groups were crowding, disabling the phenyl and the heterocyclic groups from optimizing the steric interactions of the amino acid residues of the PIM1.

The molecular docking study on MAPK14 (p38α) revealed that the three ligands can adapt their conformation in the interaction site. However, only Kaempferol was able to reach the depth of the pocket while the steric void space for the other ligands was more important (Fig. 8c).

## Discussion

After 30 years of clinical use, 5-Fluorouracil remains the cornerstone therapy for colorectal cancer. However, development of 5-FU resistant tumor cells is a limiting factor in CCR successful chemotherapy. Indeed chemo-resistant cells preserve unlimited proliferative potential, are protected from apoptosis and stimulate pathological angiogenesis, promoting then the progression of metastatic disease. Thus, novel therapeutic strategies to improve the effectiveness of 5-FU chemotherapy and to overcome drug resistance are critically needed.

One possible way to overcome or delay the emergence of resistant cancer cells is to co-administer drugs with different molecular mechanisms^37^. Combination treatments of natural polyphenols with anticancer drugs showed promising results greatly accepted by cancer researchers^38^. In the present report, we relied on our previous work in which we have shown a potent anti-tumoral effect of quince peel polyphenolic extract (Peph) through induction of apoptosis and cell cycle arrest, inhibition of angiogenic factors and improvement of therapeutic efficiency of 5-FU on human colon adenocarcinoma LS174 cells^25^. Here, we proposed to evaluate the effect of Peph extract in 5-FU-resistant colon cancer cells. For this, we established 5-FU-resistant cells (LS174-R) generated from parental sensitive colon cancer LS174 cells after continuous exposure to 60 µM of 5-FU. The 5-FU-resistant cells displayed the typical morphological changes associated to growth decrease relative to parental cells. Usually, drug-resistant cancer cells present slow growth^39^ which is in accordance with the study of Dallas *et al*.^40^ highlighting reduced proliferative rate in both 5-FU- and oxaliplatin-resistant colon cancer HT29 cells in comparison to parental one. We found that both colon cancer cells were able to form multicellular three-dimensional spheres when grown in low-adherent conditions. However, 5-FU-resistant cells produced less-condensed spheroids with a small amount of cancer cells non-incorporated into the compact cell aggregates. These cells known as non-spheroid forming (NSF) cells present loss of cell-cell adhesion associated to the inhibition of E-cadherin expression^41^. Interestingly, our result supports the reduced level of E-cadherin protein in our 5-FU-resistant cells in comparison to parental LS174 cells. The loss of E-cadherin expression promotes the epithelial-mesenchymal transition (EMT) process in epithelial tumor progression^42^. The EMT is a complex process that involves several signaling pathways, including FAK and Src activation^43^. Interestingly, our finding indicates the up-regulation of these proto-oncogenes suggesting the acquisition of more aggressive cell features in the 5-FU-resistant cancer cells.

ATP-binding cassette (ABC) transporters play a crucial role in the development of resistance by the efflux of anticancer agents outside the cancer cells^13^. Our result highlights the up-regulation of ABC sub-family G member 2 (ABCG2) and multidrug resistance-associated protein 1 (MDR1) in 5-FU-resistant LS174-R cells, which suggests that these two proteins affect the intracellular concentration of 5-FU conferring resistant phenotype to LS174-R cells.

The protection from apoptosis also constitutes an effective mechanism implicated in colorectal cancer resistance^12^. Our data showed the reduced expression of the pro-apoptotic mediator Bad in the refractive cells relative to parental one. This effect may be in part regulated by Src activation, which could reduce pro-apoptotic stimuli through the inhibition of death accelerators, such as Bad, Bax and caspase-9 proteins^44^.

Although 5-FU exerts its cytotoxic effect during the S phase of the cell cycle, prolonged G1 and S phases may provide cancer cells more time to repair the damage induced by this drug^45,46^. This correlates with the prolonged S phase and reduced proliferative rate in refractive cancer LS174-R cells in comparison to parental LS174 cells.

As comparative studies highlighted the resistant phenotype of 5-FU-resistant LS174-R cells, we studied their sensitivity to combination treatment of quince extract or its phenolic compounds with 5-Fluorouracil. Our data demonstrated that Peph extract did not affect the viability of resistant cells contrary to parental cells. Thus, we proposed to assess the effect of different phenolic compounds from total Peph extract on the viability of both sensitive LS174 cells and 5-FU-resistant LS174-R cells. We found that the thirteen phenolic compounds exhibited a dose-dependent inhibition effect on the viability of parental LS174 cells. However, Quercetin, Rutin, (+)- Catechin, (−)- Catechin, Hyperin, Isoquercitrin, Chlorogenic acid, Cryptochlorogenic acid, Neochlorogenic acid and p-coumaric acid compounds failed to induce significant inhibitory effect on the viability of 5-FU-resistant LS174-R cells. Interestingly, only Kaempferol and its analogs, Kaempferol 3-O-glucoside and Kaempferol 3-O-rutinoside were able to reduce the viability of chemo-resistant cells. Kaempferol presented the highest anti-proliferative activity with about 50% of inhibition at a concentration of 75 µM compared to its analogs that induced a slight inhibitory effect at concentration of 120 µM.

Interestingly, Kaempferol in combination with 5-FU improved the effectiveness of individual treatment and caused more decrease in colony formation of both sensitive and 5-FU-resistant cells even at low concentration (15 µM) of the compound. This chemosensitizing effect of Kaempferol to 5-FU chemotherapy highlighted an additive effect between 5-fluorouracil and Kaempferol in parental cells and synergistic interaction in 5-FU-resistant cells.

To investigate the putative cell targets of Kaempferol, we conducted a series of computational analysis. Our results suggest three intracellular protein targets CDK6, PIM1 and MAPK/p38 that have been reported to play an important role in colorectal carcinogenesis^31,47,48^. MAPK/p38 is positively correlated with typical features of cancer aggressiveness, such as migration and invasion. The p38 is highly expressed in CRC biopsies and inflammatory bowel disease-associated human CRC specimens^31^. MAPK14/p38 was shown to be inhibited by Kaempferol with an IC_50_ of 18 µM^36^.

It has been reported that the CDK6 expression increases from non-neoplastic mucosa through adenoma to submucosal invasive carcinoma to regulate the progression and metastasis of CRC^47^. CDK6 was found to interact with RB1^49^, and its expression is also controlled by the MAPK14/p38 signaling pathway^50^. CDK6 is inhibited by Kaempferol with an IC_50_ of 22 µM^51^. However, we did not detect the expression of CDK6 *in vitro*, excluding any role played by this factor in our cellular model.

Kaempferol was also identified as an inhibitor of the proto-oncogene PIM1 with an IC_50_ of 1.288 µM^52^. This Serine/threonine kinase is implicated in the cell signaling process of FOXO3 and STAT3^53^ which are members of the interaction network. PIM1 promoted the proliferation, differentiation and cell survival of colon cancer and its inhibition has been suggested as a possible target for therapeutic intervention^48^.

PIM1 could be considered as a highly potential target since Kaempferol reproduced the interaction model of Myricetin in a near exact manner. Pharmapper analysis also suggested a putative interaction of Kaempferol with MAPK14/p38 while no appearance was noticed for PIM1.

On the other hand, our docking study suggests that the glucosyl groups carried by K3g and K3r analogs of Kaempferol are too bulky to allow the fitting of the ligands inside the interaction pockets of the two putative targets. However, Kaempferol structure allows it to optimize the interactions with several amino acids including the most inner residues of the interaction site. Thus, this *in silico* study could explain why Kaempferol was endowed with the best inhibitory effect on the viability of resistant colon cancer LS174 R cells *in vitro* compared to its analogs.

Kaempferol, a natural flavonoid, is known for its antioxidant property^29^. Interestingly, this compound alone or combined with 5-FU significantly reduced ROS production in both sensitive and 5-FU-resistant cells. Over the past several years, researchers have associated ROS to malignant transformation. The generation of ROS mediated the expression of proteins involved in proliferation, tumor cell death or survival, invasion, angiogenesis, and metastasis^54^. We then tried to uncover the mechanisms of resistance to 5-FU in LS174-R cells and to shed light on the cellular targets through which Kaempferol restored 5-FU sensitivity in unresponsive LS174-R cells to drug.

The retinoblastoma protein (pRb) and p53 are known as tumor suppressor proteins that inhibit the transcription of different oncogenes involved in tumor growth and metastasis^55^. Our results suggest the acquisition of other malignant properties at least through increased phosphorylation of the protein Rb and dephosphorylation of p53 in chemo-resistant LS174-R cells. It has been reported that the hypophosphorylated form of pRb binds to the transactivation domain of E2F to inhibit cell cycle progression through the inhibition of CDK activity^56^. Interestingly, the combined treatment was effective in blocking 5-FU-resistant cells in G2/M phase which involves dephosphorylation of Rb and cdc2 (CDK1) proteins. According to our results, previous reports demonstrated that the arrest of G2/M checkpoint transition is controlled by down-regulation of cdc-family proteins^57^. In sensitive cells, our data suggest that the cooperative mechanism exploited by Kaempferol and 5-FU caused cell cycle arrest in S phase, which depends on p53, and p21 activation. Accordingly, this effect is in line with the work of Wiegering *et al*.^58^ describing the major role of several small molecules (identified from the compound library of the National Cancer Institute) in inducing p53 activation and tumor apoptosis (RITA) which in turn enhance the antiproliferative response to 5-FU and increase the expression levels of p53 protein in colorectal cancer cells.

Cell cycle arrest and apoptosis constitute the primary mechanisms that inhibit tumor formation by preventing inappropriate expansion of cells with malignant potential^59^. Acquired resistance to apoptosis represents an essential feature related to 5-FU resistance. Our finding highlights a pro-apoptotic effect of 5-FU treatment in sensitive cells without any considered activity in chemo-resistant cells. Interestingly, Kaempferol treatment increased the percentages of apoptotic cells approximately to 40% and 25% in sensitive and 5-FU resistant cells respectively. The combination of Kaempferol with 5-FU was crucial for effective sensitization of LS174-R cells to apoptosis showing that they act synergistically to trigger cell death. The increased level of PARP expression associated to the activation of caspase-3 and caspase-9 further confirmed the effectiveness of combined treatment in both parental and resistant cells in inducing apoptosis. Several therapeutics tend to eliminate transformed cells by the induction of apoptosis through activation of the effector caspases^60^. Our result suggests the therapeutic role of concomitant use of Kaempferol and 5-Fluorouracil for resistant colorectal cancer to 5-fluorouracil.

A number of signaling molecules and transcription factors such as the JAK/STAT3, Wnt/β-catenin, PI3K/Akt, MAPK and NF-κB signaling pathways are involved in the dysregulation of death machinery and progression of colorectal cancer^30^. Our results showed that MAPK (ERK1/2, p38) and PI3K/AKT (AKT) signaling pathways were activated in 5-FU-resistant LS174-R cells in comparison to the parental LS174 cells. This data suggests that acquisition of 5-FU resistance in generated 5-FU-resistant cancer cells is associated with more aggressive cell features related to the activation of AKT and ERK1/2/p38 MAPK signaling pathways. Various studies have revealed the upregulation of survival signaling pathways, including PI3K and ERK to prevent anti-cancer agents-induced cell apoptosis in multidrug resistant cancer cells^61^. The p38/MAPK pathway was also identified as a mediator of drug resistance in colorectal cancer^31^. Our finding indicated that the anti-apoptotic status of the chemo-resistant LS174-R cells to 5-FU treatment may be mediated by the intense activation of AKT and ERK/p38 MAPK pathways.

We observed that the 5-Fluorouracil treatment inhibited ERK1/2 and p38 activations in parental cells but did not affect their activity in chemo-resistant LS174-R cells. Interestingly, we found that Kaempferol alone or combined to 5-FU was able to modulate the expression of such protein kinases in both sensitive and 5-FU-resistant cells. We observed an up-regulation of the expression level of ERK1/2 associated to the inhibition of the one of p38 kinase. Generally, ERK activation has been associated to cell proliferation, differentiation and survival, but activated ERK has also been involved in growth arrest and induction of apoptotic cell death by various cytotoxic agents ^62^. Our result is in accordance with several studies that reported the activation of the MEK/ERK pathway upon the inhibition of p38/MAPK to lead to cell cycle arrest and apoptosis in colorectal cancer cells. A study by Zhang *et al*.^63^ demonstrated that, in combination with cisplatin treatment, the pharmacological inhibitor of ERK increased the phosphorylation of p38 and that p38 inhibition promoted the activation of ERK in Hela cells. The crosstalk between p38α and ERK pathway could represent a useful tool in CRC therapy^31^. Our data suggest that Kaempferol exerts its sensitizing effect through the regulation of ERK and p38 kinases. Our result also supports *in silico* analysis that explained the putative interaction of p38 and Kaempferol, which can adapt its conformation in the p38 binding site and thereby causes its inhibition.

We found that Kaempferol, by its own or combined with 5-FU, induced the inhibition of AKT along with the dephosphorylation of its target, the transcription factor FOXO3a. The hypophosphorylated active form of FOXO3a is known to bind to promoters of target genes involved in apoptosis and cell cycle arrest, inducing their transcription and thus contributed to tumor suppression^64^. The first negative control mechanism of FOXO3a is mediated by Phosphatidylinositol 3-kinase (PI3k)-activated AKT that stimulates the phosphorylation of FOXO3a promoting its interaction with nuclear export protein and its proteasomal degradation. Moreover, the study of Zhang *et al*.^63^ supported that inhibition of p38 pathway enhanced the expression of FOXO3a. The blocking of p38α-induced chemosensitization of tumor cells passes through nuclear accumulation of FOXO3a and activation of its pro-apoptotic gene targets^31^. Thus, Kaempferol inhibited the AKT and p38 kinases, activating then the tumor suppressor FOXO3a. It is well documented that compounds that reactivated FOXO3 based on its tumor suppressor property are considered as very attractive anti-cancer therapy^65^. Therefore, our finding suggests that the therapeutic potential of Kaempferol passes at least in part through the activation of FOXO3a.

Since AKT activation promotes the expression of pro-survival transcription factors and inhibits the pro-apoptotic FOXO3a^66^, we investigated the involvement of two other transcription factors, nuclear factor-κB (NF-κB) and STAT3 known to be activated in a wide variety of cancers, including colorectal cancer^67^. These regulatory proteins provide a survival mechanism by regulating the expression of target genes involved in inflammation, cell proliferation, survival, angiogenesis, invasion and metastasis, thereby representing a major causative factors for drug resistance^68,69^. Interestingly, our result indicated that Kaempferol alone or its concomitant use with 5-Fluorouracil reduced the protein level of phospho-STAT3 in both parental and chemo-resistant cells. Surprisingly, Kaempferol, 5-FU or their combined treatment induced the activation of NF-κB in sensitive LS174 cells whereas this compound alone or in combination with the drug decreased the level of its phosphorylated form in resistant LS174-R cells. In this context, Samuel *et al*.^70^ have reported that NF-κB activation varies with the cellular make up. The activation of NF-κB by several chemotherapeutic agents, including 5-Fluorouracil may be functionally uncoupled with anti-apoptotic outcomes^70,71^. In the other hand, several studies suggest the involvement of NF-κB activation in the resistance of colon cancer cells to 5-FU^72,73^. Targeting NF-κB inhibition may be used as a novel preventive and therapeutic strategy against chemoresistant human cancers^73^.

Given that cytokines and growth factors production is tightly intertwined to NF-κB and STAT3 activation^67^, we have consequently investigated the protein expression of VEGF-A and IL-8, which promote angiogenesis and pathological neovascularization in colorectal cancer^74^. Importantly, we found that the two angiogenic mediators were highly overproduced in the generated 5-FU-resistant cells in comparison to the parental LS174 cells. The VEGF is known to confer resistance to chemotherapy by inducing the expression of anti-apoptotic proteins^75^. It has been demonstrated that high levels of IL-8 are also linked to tumor development, angiogenesis, metastasis and anti-cancer drug resistance^8^. Consequently, our result suggests that acquisition of resistance to 5-FU could be related in part to up-regulation of the angiogenic factors. Interestingly, Kaempferol alone or its concomitant use with 5-FU significantly reduced the VEGF-A and IL-8 production in 5-FU-resistant LS174-R cells. However, we observed only a slight decrease in VEGF-A production without any effect on the secretion of IL-8 in parental cells. This effect could be explained by the fact that VEGF is the direct target gene of STAT3^69^. Furthermore, NF-κB activation regulates the expression of downstream target genes including cytokines (IL-6, IL-8) and growth factors (VEGF)^76^. Our data suggest that the potent chemosensitizing effect of Kaempferol in our chemo-resistant cells passes through the inhibition of NF-κB and STAT3 transcription factors, leading then to VEGF-A and IL-8 downregulation.

The conversion of 5-Fluorouracil to active metabolites passes through several enzymatic steps^77^. Previous studies have reported the closely relationship between 5-FU metabolism and drug resistance^78^. Then, to better identify the mechanism by which Kaempferol sensitizes chemo-resistant LS174-R cells to 5-FU, we analyzed the expression of five genes involved in the metabolic pathways of 5-FU such as TS, TK, FPGS, DHFR and DPD. Our result showed that acquired resistance in the 5-FU-resistant cells was associated with significant increase in the mRNA levels of these enzymes, especially, for TS (with 91.77 fold induction). Only the DPD, implicated in 5-FU catabolism, was slightly decreased in comparison with parental cells. This result was confirmed by western blotting analysis for TS and TK proteins levels. The overexpression of TS is one of the most well-known mechanisms of acquired resistance after 5-FU exposure^79^. Previous study indicated that low expression of TS in CRC patients was associated with improved median survival compared with those with higher levels of TS enzyme^77^. The overexpression of TK also represents a potential mechanism of 5-FU resistance^80^. Interestingly, Kaempferol alone or combined with 5-FU significantly decreased the mRNA expression of the five genes implicated in 5-FU metabolism only in chemo-resistant LS174-R cells. This effect was associated with the inhibition of the expression of TS and TK proteins, which play a key role in dTMP (deoxythymidine monophosphate) formation, essential for DNA synthesis and cellular proliferation. Our result is in line with the study of De la Cueva *et al*.^81^ that reported that down modulation of TS and TK mRNA and protein levels affects the mechanisms of resistance in 5-FU-resistant colon cancer cells through dephosphorylation of Rb protein associated to E2F1 inhibition. Further investigation also revealed that PI3K/AKT activation induced the overexpression of TS in CRC cells resistant to 5-FU chemotherapy^82^. In addition, the low expressions of TS, TK, DHFR and DPD were linked to tumor progression in CRC patients.

The present study provides evidence for the chemosensitizing effect of Kaempferol to 5-FU chemotherapy of LS174-R cells by exerting synergistic inhibitory effect through blocking the production of ROS and modulating JAK/STAT3, MAPK, PI3K/AKT and NF-κB signaling pathway, involved in the progression and development of colorectal cancer. Kaempferol alone or in combination with 5-FU decreased the production of IL-8 and VEGF-A and reduced the expression of five genes involved in 5-FU metabolism in chemo-resistant cells. Taken together, our results suggest a potential chemotherapeutic role of Kaempferol, which could represent a novel concept for overcoming 5-FU resistance in patients diagnosed with colon cancer. Moreover, pharmacokinetic and pharmacodynamics studies demonstrated that Kaempferol is a non-toxic natural agent, which is safe *in vivo* and bioavailable^83^.

Although our results are encouraging, our resistant cell model did not take into account the presence *in vivo* of multiple cell types in addition to the complexity of the tumor environment where new interactions could be involved in the resistance of colorectal cancers. Further investigation is warranted to better verify if the *in vitro* chemosensitizing effect of Kampferol in 5-FU-resistant LS174-R colon cancer cells can be extended to the *in vivo* setting.

## Materials and Methods

### Quince peel polyphenolic compounds

Quince peel polyphenolic compounds identified in the total polyphenolic extract (Peph) [Quercetin (Q), Rutin (R), (+)-Catechin (+C), (−)-Catechin (−C), Hyperin (H), Isoquercitrin (I), Chlorogenic acid (ChA), Cryptochlorogenic acid (CrA), Neochlorogenic acid (NeA), p-coumaric acid (PcA), Kaempferol (Kaemp), Kaempferol-3-O-glucoside (K3g) and Kaempferol-3-O-rutinoside (K3r)] were purchased from Sigma (Sigma-Aldrich, St. Louis, MO).

### Cell culture and generation of 5-FU-Resistant Colon Cancer Cells

Human colon adenocarcinoma LS174 cell line (CL-188), were obtained from American Type Culture Collection (ATCC, Manassas, VA). The cells were cultured in DMEM (Dulbecco’s Modified Eagle’s Medium) supplemented with 10% fetal bovine serum (FBS; GIBCO) and 50 U/ml penicillin and 50 µg/ml streptomycin. The 5-FU-Resistant cells maintained in 100 mm^2^ petri dishes were generated by continuous exposure of LS174 cells to increasing doses of 5-FU (10–100 μM). Medium was changed three times a week and adherent cells were passaged using trypsin/EDTA. Afterwards, the surviving 5-FU-resistant cells were maintained in complete culture medium containing 60 μM of 5-Fluorouracil.

### Cell viability

The growth of parental LS174 cells and 5-FU-resistant cells was assessed by 3-(4,5-dimethylthiazol-2yl)-2,5-diphenyltetrazolium bromide (MTT) assay. Cells were seeded in 96-well plates (1000 cells/well) for 24 h and then incubated for 72 h in the presence of vehicle (control) and testing agents. At the end of the treatments, 50 µl of MTT solution (1 mg/ml final) were added into each 96-well plate and the cells were incubated for a further 3 h at 37 °C. Thereafter, the medium was removed and 100 µl of dimethyl sulfoxide (DMSO) was added to each well to dissolve the formazan crystals. The optical density (OD) at 540 nm was measured with a microplate reader (MULTISKAN, Labsystems). The cell viability was expressed as percentage of the viable cell number in treated cells relative to mock-treated cells (control). Cell viability was calculated using the following formula:

$$ \% \,{\rm{cell}}\,{\rm{viability}}={{\rm{OD}}}_{{\rm{Test}}}/{{\rm{OD}}}_{{\rm{Control}}}\times 100.$$

### Colony formation assay

Parental and resistant cells were seeded in six-well plates (2 × 10^5^ cells/well). After 24 h, cells were treated with 15 µM or 75 µM of Kaempferol alone or combined to 60 μM of 5-Fluorouracil. After 72 h of treatment, the medium was removed and cells were trypsinized and plated at low density of 2000 cells per six-well plate. Cells were then cultivated for 10 days. Colonies were stained with crystal violet and clones for each condition were photographed. The number of colonies was scored by CFU scope quantification software. Results are expressed as the number of colony forming cells per well in percentage and normalized to control (vehicle, considered to represent 100%).

### Spheroid generation

The colon cancer cells LS174 and LS174-R were analyzed for spheroid formation capacity in ultra-low attachment (ULA) round bottom 96-well plates. Each well was coated with 100 µl of agarose. After 30 min at 37 °C, cells were seeded (1000 cells/well) for 5 days under standard conditions (37 °C, 5% CO_2_). After the incubation period, cells were photographed under light microscopy.

### Sensitization assay

Both colon cancer cells were seeded in 96-well culture plates (1000 cells/well). After 24 h, the cells were treated with serial concentrations of Kaempferol (1, 5, 10, 15, 30, 60, 75 µM) for various periods (8 h, 12 h and 24 h). Cells were then exposed to 60 µM of 5-FU after removing and/or maintaining Kaempferol in cell culture medium. After 72 h of treatment, the viability of parental and 5-FU-resistant cells was determined by MTT assay as described above. The drug concentration that resulted in a 50% growth inhibition (GI50) was determined graphically from sigmoidal dose-response curves.

### Drug and compound interaction: Determination of the Combination Index (CI) value

Parental and resistant cancer cells were seeded at 1000 cells/100 µl in 96-well plates and allowed to attach for 24 h. Cells were treated for 72 h with increasing concentrations of Kaempferol alone, 5-FU alone and the combination of both. Concentrations for drug combination studies were based on IC50 values (IC50/4, IC50/2, IC50, IC50*2, IC50*4). Following indicated treatments, the viability of both colon cancer cells was determined by MTT assay as described above. The interaction between 5-FU and Kaempferol was determined after calculating the Combination Index values, where a CI < 1 indicates synergism, CI = 1 additive effect and CI > 1 antagonism^84^.

The Combination index (CI) values were calculated based on the following formula:

$${\rm{CI}}={{\rm{C}}}_{{\rm{A}},{\rm{X}}}\,{/\mathrm{IC}}_{{\rm{X}},{\rm{A}}}+{{\rm{C}}}_{{\rm{A}},{\rm{X}}}\,{/\mathrm{IC}}_{{\rm{X}},{\rm{A}}}$$

C_A,X_: the concentrations of drug A to produce the given effect (e.g., IC50).

IC_X,A_: the concentrations of drug A and B in combination to provide the same effect (e.g., IC50).

### Measurement of reactive oxygen species (ROS)

The intracellular ROS assay employs a cell-permeable fluorogenic probe, CMH2DCF-DA (life technologies, Oregon, USA). This molecule passively diffuses into cells and is deacetylated by cellular esterases to non-fluorescent 2′,7′-dichlorodihydrofluorescin (DCF), which is rapidly oxidized to highly fluorescent adduct in the presence of ROS. Colon cancer cells were cultured in 96-well plates (2000 cells/well) and treated with the listed agents for 72 h. Cells were washed with PBS (1X), resuspended in HBSS (GIBCO) and then incubated with 10 µM of CMH2DCFDA at 37 °C for 30 min in dark and in a CO_2_ incubator. Fluorescence was measured by the Varioskan Flash microplate reader (Thermo Scientific) with an excitation and emission wavelengths of 492 and 517 nm, respectively.

### Cell cycle analysis

Sensitive and chemo-resistant colon cancer cells were seeded into six-well plates and then exposed for 72 h to testing agents. Thereafter, cells were harvested by trypsinization and fixed with 70% ice-cold ethanol. After washing twice with 1X ice-cold PBS, cells were resuspended in propidium iodide (PI)/RNase staining solution (Cell Signaling Technology; Danvers, MA) for 30 min in the dark at 37 °C. Cell cycle progression was analyzed on a Becton–Dickinson FACScanto II flow cytometer and further analyzed with BD FACSDiva 6 software (Becton–Dickinson). The PI fluorescence signal at FL2-A peak versus counts was used to determine cell cycle distribution and the data were analyzed using the Modfit software.

### Assessment of Apoptosis

Apoptosis was quantitatively assessed using the annexin V/PE apoptosis detection kit (BD-Pharmingen) according to the manufacturer’s protocol. Briefly, the cells treated with the molecules of interest or vehicle as a negative control were collected by centrifugation, washed with PBS (1X) and resuspended in 100 μl of binding buffer (1X). Thereafter, cells were treated with 4 μl of Annexin V conjugated to phycoerythrin (PE) and 4 µl of 7-AAD and then rapidly incubated in the dark for 15 min. Stained cells were analyzed on a Becton–Dickinson FACScanto II flow cytometer and further analyzed with BD FACSDiva 6 software (Becton–Dickinson). Cell death was quantitatively evaluated by measuring the proportion of annexin V-positive cells, regardless of their staining for 7-AAD in order to include both early apoptotic and dead cells. Values are given in percent of total cell number. Percentage of apoptotic cells (%) was calculated as follows: early apoptotic cells (%) + late apoptotic cells (%).

### Western blotting analysis

After 72 h of treatment with the molecules of interest, cells were solubilized in 100 µl of Laemmli buffer (1X) at room temperature. Protein content of the cell lysates was quantified using the BCA method (Bicinchoninic Acid Protein Assay kit, Sigma). Equal amounts of protein (30 µg/sample) were resolved on sodium dodecyl sulfate polyacrylamide gels (SDS-PAGE). After electrophoresis, proteins were transferred onto polyvinylidene difluoride (PVDF) membrane (Immobilon-Millipore) and incubated for 1 h at room temperature with blocking buffer (5% non fat dry milk). The membranes were probed overnight with primary antibodies (1:1,000 dilutions): [ABCG2 (MAB4146) was obtained from Millipore. β-actin, MDR1, cleaved PARP, cleaved caspase 3, cleaved caspase 9, Bcl-2, Bad, E-cadherin, vimentin, β-catenin, c-Src, FAK, p53, phospho p53 (Ser 15), phospho-FOXO3a, phospho-Stat3, phospho-ERK_1/2_, phospho-p38, phospho-AKT, phospho-NF-*κ*B, Thymidylate Synthase and Thymidine Kinase were obtained from Cell Signaling Technology (Danvers, MA)] and incubated with a horseradish peroxidase-conjugated anti-IgG (Promega, Madison, WI) in a blocking buffer for 1 h. After washing, the blots were developed with enhanced chemiluminescence (ECL) (Millipore) and exposed to X-ray film.

### Real time quantitative RT-PCR

To assess the expression of several genes involved in 5-FU metabolism, total RNA was isolated from both sensitive and 5-FU-resistant colon cancer cells after 72 h of treatment with molecules of interest. Reverse transcription was realized with 1 µg of RNA from each sample. After cDNA preparation, real-time PCR was carried out using the LightCycler System (Roche Applied Science, Mannheim, Germany). PCR was set up at 2.5 mM MgCl_2_, 10 μM of each primer (Eurofins Genomics, USA), 4 μl of recover DNA and 5 µl of Master Mix (KAPA Biosystems) in a final volume of 10 μl. Data analysis was essentially performed using “Fit Point Method” in the LightCycler software version 3.5.3. GAPDH gene was used as the internal reference.

To calculate the relative expression of transcripts, the 2^[−ΔΔC (T)]^ method was used^85^. Each calculated fold was determined from the average of 3 independent experiments run in duplicate. The sequences of PCR primers are available upon request.

### Determination of cytokines concentration

Seventy-two hours post-treatment with the testing agents, cell culture supernatants from parental LS174 cells and 5-FU-resistant cells were centrifuged at 1000 rpm for 5 min and the cells were counted. The VEGF-A and IL-8 concentrations were quantified using an ELISA kit Quantikine human Immunoassay (Thermo SCIENTIFIC) following the manufacturer’s guidelines and normalized to cell number.

### *In silico* analysis of Kaempferol interaction with the cellular molecular targets

To get insight about the interaction network within which the Thymidylate synthase exerts its function, we retrieved the accession numbers of all the proteins whose expression was evaluated by western blot (FOXO3a: O43524, NF-κb1: P19838, STAT3: P40763, P53: P04637, phospho-rb: P06400, AKT: P31749, ERK (MAK1): P28482, Thymidylate synthase: P04818, MAPK14 (p38 14): Q16539, MAPK10 (p38 10): P53779). The list of the proteins was submitted to the NetworkAnalyst server^86^. The analysis was performed based on the protein-protein interaction data of the InnateDB^87^, which compiles more than 136000 experimentally validated interactions representing 3000 pathways from human, mouse and bovine genes.

### Ligands structure

The 3D coordinates of Kaempferol (ChEMBL150) and Kaempferol-3-O-glucoside (ChEMBL453290) were obtained from the ChEMBL database. Those of Kaempferol-3-O-rutinoside were obtained from the ChEBI database using the identifier ChEBI69657. All the structures were converted to the SYBYL mol2 file format.

### Prediction of cellular target by pharmacophore mapping

Given a ligand structure, the PharmMapper server^88^ assigns its pharmacophore representation to identify a list of potential protein targets by using the reverse pharmacophore mapping approach. The Kaempferol coordinates were first submitted to the PharMapper server which generates multiple conformations for the ligand structure. The pharmacophore models of each conformation of the ligand and those of the protein targets (considering only the human protein targets: 2241 proteins) were then aligned against each other. A score was then calculated describing the pairwise fit level between the pharmacophore models of the ligand and each target protein in the database. A report was finally generated in which all the potential targets are listed according to their fit score.

### Molecular docking

The molecular docking of Kaempferol, Kaempferol-3-O-rutinoside and Kaempferol-3-O-glucoside were processed with the program PLANTS (Protein-Ligand ANT System)^89^. The ligands and the receptors were first treated with SPORES^90,91^ to generate the topologies of the interacting partners affecting the hydrogen atoms and assigning the hybridization states of the atoms. PLANTS uses the so called colony optimization algorithms to sample the rotational and translational degrees of freedom of the ligand and search for a low energy configuration. The internal ligand flexibility was also modeled by sampling the rotational bonds degrees of freedom. The docking domain was set to a sphere of 10 Å radius centered at the catalytic binding site of the target proteins. The software returned an ensemble of putative complexes, which were then clustered to account for the geometrical similarity based on a cutoff difference of 2 Å. The putative docking solutions were ranked according to the chemPLP scoring function, which was dimensionless. The lowest values correspond to the most favorable docking solutions.

### Statistical analysis

Data from individual experiments are expressed as means ± S.E. Differences between means were evaluated using Student’s *t*-test. Differences were considered to be statistically significant at *P* < 0.05.

## Electronic supplementary material

Supplementary figures S1 and S2
