# Supplementary material for: The Phenolic compound Kaempferol overcomes 5-fluorouracil resistance in human resistant LS174 colon cancer cells
Source: Sci Rep. 2019 Jan 17;9:195. doi: 10.1038/s41598-018-36808-z (PMC6336835; doi:10.1038/s41598-018-36808-z)
Supplement: Supplementary file 1 — Supplementary figures S1 and S2 [file 41598_2018_36808_MOESM1_ESM.pdf]

The Phenolic compound Kaempferol overcomes 5-fluorouracil resistance in human resistant LS174 colon cancer cells

Ichrak Riahi-Chebbi[1,5], Soumaya Souid[1,5§], Houcemeddine Othman[2,5§], Meriam Haoues[3,5], Habib Karoui[1,5], Alain Morel[4], Najet Srairi-Abid[2,5], Makram Essafi[3,5] and Khadija Essafi-Benkhadir[1,5*]

[1]Institut Pasteur de Tunis, LR11IPT04/LR16IPT04 Epidémiologie Moléculaire et Pathologie Expérimentale appliquée aux Maladies infectieuses, 1002, Tunis, Tunisia.

[2]Institut Pasteur de Tunis, LR11IPT08/LR16IPT08 Venins et Biomolécules thérapeutiques, 1002, Tunis, Tunisia.

[3]Institut Pasteur de Tunis, Laboratoire de Recherche sur la Transmission, le Contrôle et l'Immunobiologie des Infections, LR11 IPT02, 1002 Tunis, Tunisia.

[4]CRCINA, INSERM, université de Nantes, université d'Angers, Paul Papin ICO cancer center, Angers, France

[5]Université de Tunis El Manar, 1068, Tunis, Tunisia.

§ These authors contribute equally to this work.

*Corresponding author: Essafi-Benkhadir Khadija, Laboratoire d'Epidémiologie Moléculaire et de Pathologie Expérimentale Appliquée Aux Maladies Infectieuses (LR11IPT04/LR16IPT04), Institut Pasteur de Tunis, 13 Place Pasteur BP 74, 1002 Tunisï Belvédère, Tunisia. E-mail: essafi.khadija@pasteur.rns.tn Tel: (+216)71783022, ext: 592 Fax: (+216)71791833.

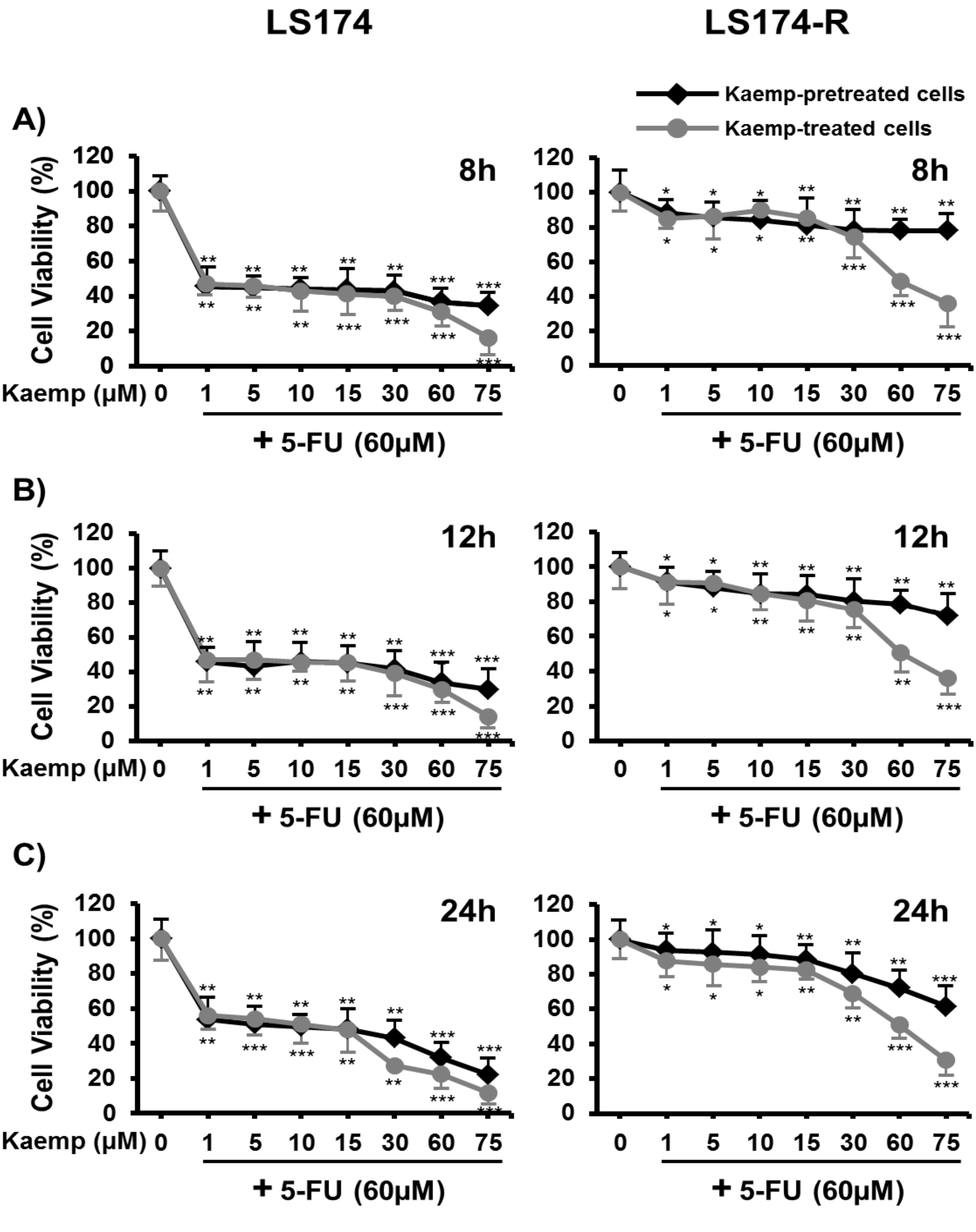

Fig. S1. Kaempferol pretreatment chemo-sensitizes resistant colon cancer cells to 5-FU chemotherapy. Both parental LS174 cells and 5-FU-resistant LS174-R cells were seeded in 96-well plates and pretreated for different time **A)** 8 h, **B)** 12 h and **C)** 24 h with increasing concentrations of Kaempferol (1-75 µM) and then exposed to 60 µM of 5-FU after removing or preserving the phenolic compound in the culture medium for additional 72 h. Cell viability was measured by MTT assay. The absorbance was measured at 540 nm. *p < 0.05, **p < 0.01 and ***p < 0.005 when compared to their respective CN.

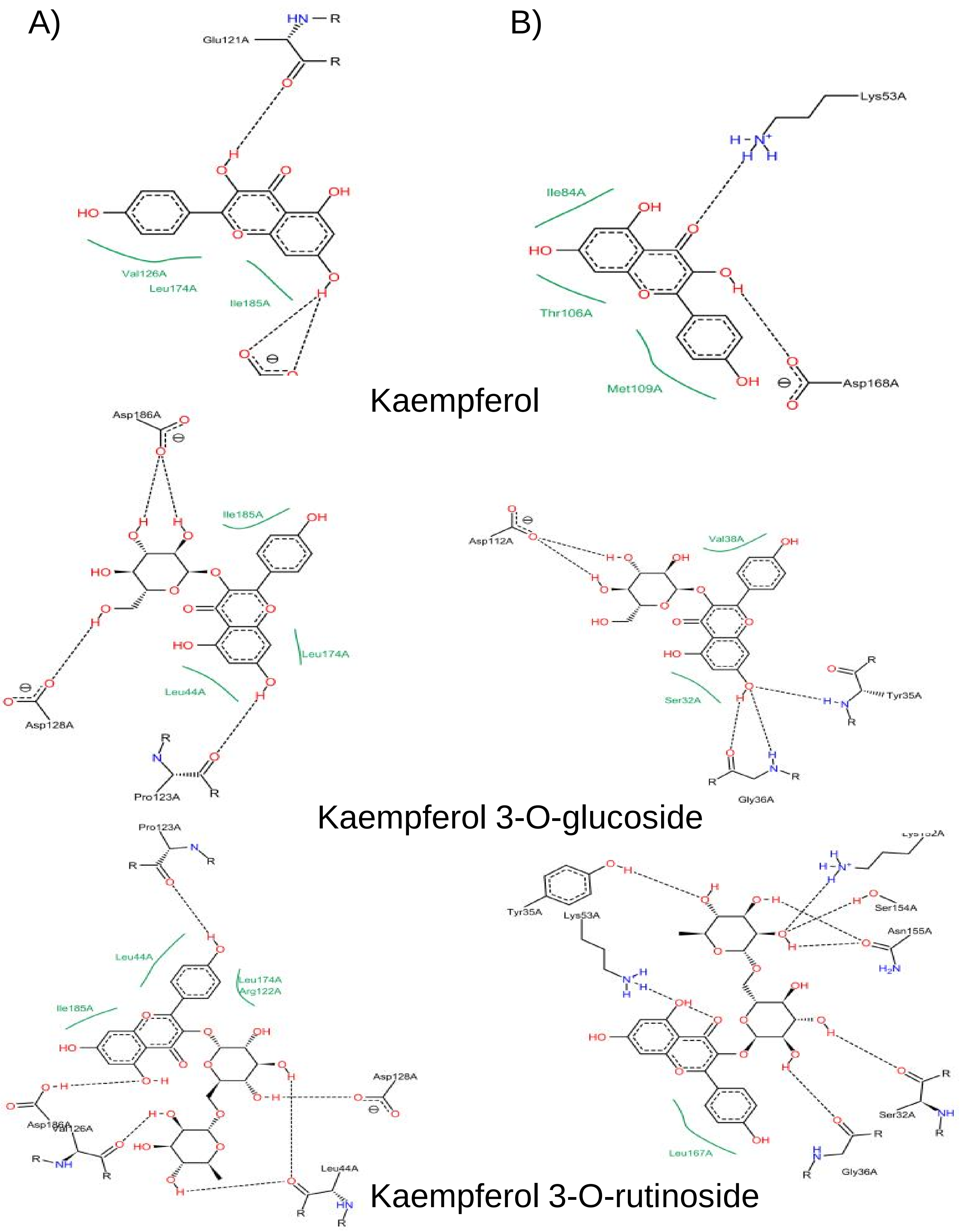

Fig.S2. Description of the interactions established by Kaempferol, Kaempferol 3-O-glucoside and Kaempferol 3-O-rutinoside with PMI1 **(A)** and p38 **(B)**. The figure was generated using PoseView (Stierand and Rarey, 2010, doi: 10.1021/ml100164p)
